# Determinants of food preparation and hygiene practices among caregivers of children under two in Western Kenya: a formative research study

**DOI:** 10.1186/s12889-022-14259-6

**Published:** 2022-10-06

**Authors:** Emily A. Ogutu, Anna Ellis, Katie C. Rodriguez, Bethany A. Caruso, Emilie E. McClintic, Sandra Gómez Ventura, Kimberly R. J. Arriola, Alysse J. Kowalski, Molly Linabarger, Breanna K. Wodnik, Amy Webb-Girard, Richard Muga, Matthew C. Freeman

**Affiliations:** 1 Gangarosa Department of Environmental Health, Rollins School of Public Health, Emory University, 1518 Clifton Road NE, Atlanta, GA 30322 USA; 2 Hubert Department of Global Health, Rollins School of Public Health, Emory University, Atlanta, GA USA; 3 Department of Behavioral, Social, and Health Education Sciences, Rollins School of Public Health, Emory University, Atlanta, GA USA; 4 James T. Laney School of Graduate Studies, Emory University, Atlanta, GA USA; 5 Uzima University College, Kisumu, Kenya

**Keywords:** Behavior change, COM-B, Intervention development, WASH, Qualitative methods, Handwashing, Food hygiene

## Abstract

**Introduction:**

Diarrhea is a leading cause of child morbidity and mortality worldwide and is linked to early childhood stunting. Food contamination from improper preparation and hygiene practices is an important transmission pathway for exposure to enteric pathogens. Understanding the barriers and facilitators to hygienic food preparation can inform interventions to improve food hygiene. We explored food preparation and hygiene determinants including food-related handwashing habits, meal preparation, cooking practices, and food storage among caregivers of children under age two in Western Kenya.

**Methods:**

We used the Capabilities, Opportunities, and Motivations model for Behavior Change (COM-B) framework in tool development and analysis. We conducted 24 focus group discussions with mothers (*N* = 12), fathers (*N* = 6), and grandmothers (*N* = 6); 29 key informant interviews with community stakeholders including implementing partners and religious and community leaders; and 24 household observations. We mapped the qualitative and observational data onto the COM-B framework to understand caregivers’ facilitators and barriers to food preparation and hygiene practices.

**Results:**

Facilitators and barriers to food hygiene and preparation practices were found across the COM-B domains. Caregivers had the capability to wash their hands at critical times; wash, cook, and cover food; and clean and dry utensils. Barriers to food hygiene and preparation practices included lack of psychological capability, for instance, caregivers’ lack of knowledge of critical times for handwashing, lack of perceived importance of washing some foods before eating, and not knowing the risks of storing food for more than four hours without refrigerating and reheating. Other barriers were opportunity-related, including lack of resources (soap, water, firewood) and an enabling environment (monetary decision-making power, social support). Competing priorities, socio-cultural norms, religion, and time constraints due to work hindered the practice of optimal food hygiene and preparation behaviors.

**Conclusion:**

Food hygiene is an underexplored, but potentially critical, behavior to mitigate fecal pathogen exposure for young children. Our study revealed several knowledge and opportunity barriers that could be integrated into interventions to enhance food hygiene.

**Supplementary Information:**

The online version contains supplementary material available at 10.1186/s12889-022-14259-6.

## Introduction

In low-income contexts, malnutrition is a critical factor in the morbidity and mortality of children under five years. 45% of all deaths of children under five in low and middle-income countries are linked to undernutrition, and 61.4 million children in Africa were stunted in 2020 [1]. Poor water, sanitation, and hygiene practices, including food hygiene, contribute to poor childhood nutrition through the ingestion of microbes that cause diarrhea. Exposure to food contaminants can occur due to inadequate handwashing habits, food handling, preparation, storage, and oversites during cultivation, harvest, and transportation to the household [2–4]. Unsafe food can contain harmful bacteria, viruses, parasites, and chemical substances which cause many diseases including diarrhea [5]. Since bacteria, viruses, and parasites are invisible, people may disbelieve their existence, negatively affecting behaviors related to optimal food and hygiene habits. Food preparation actions to prevent foodborne contamination include thorough initial cooking and reheating of food, in terms of both temperature and time; limiting the time cooked food is stored at ambient temperature to less than 4 h; washing utensils; and handwashing with soap before and during food preparation and before feeding children [6, 7]. The transfer of pathogens into prepared meals is exacerbated by a lack of thoroughly washing contaminated hands after defecation of the child and caregiver, and after cleaning areas and items touched by child feces, as well as the lack of cleaning utensils used before, during and after meal preparation [8]. Some barriers preventing cleaning are difficult-to-clean household surfaces water scarcity [9]. Food can become contaminated through high ambient storage temperatures, lack of refrigeration, poor food storage facilities, environmental fecal contamination, and too low temperature. Cooking fuel scarcity may lead to not thoroughly cooking and reheating food [9–11]. Women may not prioritize optimal hygienic food preparation and safety because of otherwise heavy workloads, poor or inadequate knowledge and ways to share about the importance of safe hygiene, correct sanitation, and hygienic food preparation practices [10]. Focusing on food hygiene practices that involve certain measures necessary for the safety of food from production to consumption can contribute to addressing these factors.

Poor sanitation is associated with the transmission of diarrheal diseases such as cholera and dysentery, typhoid, intestinal worm infections, and polio. Inadequate food hygiene is considered a major contributor to the transmission of enteric pathogens, though good estimates of the contribution of foodborne diarrheal infections and other downstream sequelae are not available [11, 12]. Diarrhea is one of the most important infectious disease determinants of stunting and is a leading cause of child mortality and morbidity worldwide, accounting for 8% of all deaths among children under 5 [13, 14]. Yet even asymptomatic infection can lead to environmental enteropathy, resulting in growth shortfalls [15, 16]. Since patients will be requested to use antibiotics when suffering from diarrhea infections, the frequent use of antibiotics can contribute to antimicrobial resistance [17]. Environmental enteropathy leaves children chronically fighting low-grade infection due to continued exposure to enteric pathogens through poor sanitation conditions. This exhausts children’s nutrient supply from their diet, impeding physical growth and development [18, 19].

Stunting remains an important public health issue in low and middle-income countries, especially in Sub-Saharan Africa [13, 20–24]. In 2014, Kenya had a 26% rate of stunting in children under 5, and the highest stunting rate of 34% in children 18–24 months; [25] the Government of Kenya has targeted a reduction of stunting rate to 14.7% by 2030 [26]. Through devolution and partnerships with non-governmental organizations, private sector and civil societies, Kenya has worked to strengthen the systems that ensure sufficient water and sanitation service delivery to improve well-being of its residents [27]. The country has supported development and implementation of guidelines targeting water, sanitation, hygiene and nutrition interventions [28]. The cycle of chronic under-nutrition and infection, often manifesting as stunting, can have major implications for long-term health and development, including learning difficulties, language domains, social-emotional functioning, physical well-being, and barriers to community participation [29–31]. Stunting is largely irreversible after the first 1000 days, leading to an intergenerational cycle of poor growth and development [32]. The first 1000 days- the period from conception to the child’s second birthday, is a crucial period for optimum health, growth, and neurodevelopment [33]. Women who were stunted in childhood remain stunted as adults and tend to have stunted offspring [14, 32] .

Interventions that combine knowledge with behavior change theories and techniques have been effective at changing behaviors related to food hygiene in high-income countries [10, 34]. However, few studies have focused on efforts on how to improve food hygiene behaviors in household environments in low-income settings [35]. Approaches like Hazard Analysis Critical Control Point (HACCP), which identify points where control measures would be effective to facilitate appropriate targeting of resources, and the Risk, Attitude, Norms, Ability, and Self-Regulation (RANAS) model which assesses contextual and psychosocial factors associated with food hygiene practices, among others, have been used [10, 36–38]. A prior study in Kenya applied Behavior Centered Design (BCD) to an intervention; BCD posits that behavior change is likely if an intervention can change the behavioral setting and cognitive processes associated with that behavior [35]. These interventions focused on specific behaviors of interest and how they contribute to food hygiene practices but have inadequately shown the interaction and influence of the combined food hygiene practices. Although these studies were conducted in peri-urban and rural communities and were successful in improving food hygiene practices of interest; their focus was limited to specific behaviors in parts, including cooking and reheating food, cleaning utensils, and handwashing, but not multiple food preparations and hygiene practices. These studies assessed the psychological factors and emotional motivators, [10, 11, 33] however, they did not often assess how opportunity factors could inhibit behaviors. While capability and opportunity gaps to practice food hygiene behaviors have often been reported, studies did not clarify how these could be overcome [39, 40].

Using the Capabilities, Opportunities, and Motivation to Behavior (COM-B) model that explored the barriers to and facilitators of optimal food hygiene and preparation practices, this study reports the formative process that informed a Catholic Relief Services (CRS) funded THRIVE II program. The goal of THRIVE II was to create a culture of care and support for HIV- and AIDS-affected children under 2 (CU2) and their caregivers in Kenya, Tanzania, and Malawi by providing ongoing support to caregivers of CU2 to practice early childhood stimulation, positive parenting, optimal infant and young child feeding, and water, sanitation and hygiene (WASH) behaviors [41]. In 2016, CRS partnered with Emory University and Uzima University (Kenya) to design an integrated WASH and nutrition behavior change intervention to be nested within a selection of THRIVE II communities to decrease stunting among CU2 [7, 41, 42]. To inform this intervention, we conducted qualitative research between August and December 2016 with caregivers of CU2 in Migori and Homa Bay counties, Western Kenya [7, 42, 43].

This study applied a theory-informed approach to explore the drivers and barriers to optimal food preparation and hygiene practices among caregivers of CU2 in Western Kenya. Our findings aim to inform the development of targeted improvements to a Care Group model an approach that uses a cadre of paid workers as facilitators, who impart knowledge and training to groups of ~ 12 volunteers (the Care Group); each volunteer is responsible to share the same knowledge and training with 10–15 local households [44] intervention in Western Kenya [41, 42].

## Methods

### Study sites and population

This research took place in six communities in Migori and Homa Bay counties that were participating in THRIVE II. The THRIVE II program was an early childhood development (ECD) program led by Catholic Relief Services (CRS) and local implementing partners, Homa Hills Community Development Organization (HHCDO) and Mercy Orphans Support Group (MOSGUP). The THRIVE II program continued and improved on previous CRS programming, THRIVE. THRIVE II aimed to support children in reaching their developmental milestones. Specifically, THRIVE II used the care group model to target children particularly at risk of not receiving ECD services because of poverty and HIV [45]. Homa Bay and Migori counties were covered by THRIVE II since they had the highest HIV prevalence in Kenya in 2016; in 2017, HIV prevalence in Homa Bay and Migori were 20.7% and 13.3% respectively, far higher than the national prevalence of 4.9% [46].

We purposively sampled THRIVE II participants for participation in the qualitative research from six health facility catchments (*N* = 3, Migori county; *N* = 3, Homa Bay county) in 2016. Preference was accorded to communities with variability in the agro-ecological zone, distance to the nearest health facility, and distance to the nearest urban center [7]. Participants were recruited from THRIVE II communities which had a minimum of six women that lived near the health facilities and were either pregnant or had CU2. Recruited religious and community leaders were identified by CRS, based on their knowledge and experiences of their specific communities. The community health workers and community health volunteers recruited for participation were based in the health facility catchment area.

### Theoretical approach

The Capabilities, Opportunities, and Motivations to Behavior (COM-B) model was used to guide the prioritization and analysis of barriers and facilitators to optimal food preparation and hygiene practices. The COM-B model is used to identify and understand determinants of behaviors and what needs to be altered to facilitate behavior change. The COM-B model focuses on three essential determinant domains necessary for practicing specific behaviors: capability, opportunity, and motivation (Table 1). The COM-B model posits that both capability and opportunity are prerequisites for motivation: people must have the physical and psychological capability to perform the behavior, the physical and social opportunity to do the behavior, and the automatic and reflective motivation to practice the behavior over other competing priorities [7].

**Table 1:** Capability, opportunity, motivation, and behavior definitions [47]

**COM-B Behavioral determinant**	**Definition**
**Capability**	Capability is an attribute of a person that together with opportunity makes a behavior possible or facilitates it
**Psychological capability**	A capability that involves a person’s mental functioning (e.g. understanding and memory)
**Physical capability**	A capability that involves a person’s physique and musculoskeletal functioning (e.g. brain and extremity)
**Opportunity**	An attribute of an environmental system that together with capability makes a behavior possible or facilitates it
**Social opportunity**	An opportunity that involves other people and organizations (e.g. social and cultural norms)
**Physical opportunity**	An opportunity that involves inanimate parts of the environmental system and time (e.g. financial and material resources)
**Motivation**	All brain processes that energize and direct behavior
**Reflective motivation**	The motivation that involves conscious thought processes (e.g. evaluations and plans)
**Automatic motivation**	The motivation that involves habitual, instinctive, drive related, and affective processes (e.g. desires and habits)

We categorized the barriers and facilitators to food preparation and hygiene practices based on COM B framework. We mapped these determinants to five mealtime behaviors which necessitate optimal food preparation and hygiene practices- food preparation and handling practices that have the potential to minimize contamination of food by pathogenic organisms. We provided operational definition of these behaviors.

### Data collection

Qualitative data were collected from October to December 2016 using focus group discussions (FGDs), key informant interviews (KIIs), and household observations. Seven research assistants from communities in western Kenya were trained over two weeks on qualitative research methods, research ethics, and data management prior to data collection. Training of the research assistants was conducted by the field team manager, a Kenyan native (EAO), with the support of a research manager from Emory University (AE). Qualifications of research assistants were: 1) Fluent Luo, Kiswahili, and English speakers; 2) experience in qualitative data collection; and 3) understanding of the study area. Kenya CRS personnel and research assistants provided input on adaptations to translation, cultural appropriateness, and length of tools (FGD and KII guides, observation checklist). Research tools were piloted with THRIVE II participants and community health workers in Migori County, researchers provided feedback to adjust tools to improve clarity and focus on thematic domains.

#### Focus group discussions

We conducted 24 FGDs with: pregnant women and mothers (*N* = 12), fathers (*N* = 6), and grandmothers (*N* = 6) of CU2 to understand their practices related to food hygiene and preparation. All participants had to be 18 years or older, and a caregiver for a child between the ages of 1-and 24 months, or a woman who identified as pregnant. Women who identified as pregnant and mothers of CU2 were selected based on their participation in THRIVE II; grandmothers had to have at least one grandchild under two years; fathers had to have at least one child under two years and were related to the THRIVE II participants [7]. Since programming was to take place over two years, pregnant women were included as they would eventually be caregivers of CU2, and their nutrition and WASH behaviors during pregnancy could affect the infant’s growth and development [48]. Six to eight participants were recruited by implementing partner members for each focus group, based on their availability and willingness to participate; 139 total participants were engaged. Keeping FGD participant numbers from six to eight provided time and opportunity for each participant to engage in the discussions.

We conducted more FGDs with mothers as primary caregivers; grandmothers and fathers were included as they may be primary or secondary caregivers, and their support, knowledge, availability, and practices can influence the behavior of the mothers. FGDs with pregnant women and mothers of CU2, and grandmothers focused on nutrition, feeding, and WASH and FGDs with fathers focused on WASH. FGDs with pregnant women, mothers and grandmothers were facilitated by female research assistants and were held in community churches or health facilities.

#### Key informant interviews

A total of 29 KIIs were conducted with religious and community leaders (*N* = 11), community health workers (*N* = 5), community health volunteers (*N* = 6), and THRIVE II staff and implementing partner staff (*N* = 7) to understand what influences food hygiene and preparation, infant and young child feeding practices, and intervention implementation. The key informants identified the determinants of community infant and young child feeding (IYCF) and WASH behaviors, based on their roles and responsibilities in encouraging optimal behaviors, leading to their recommendations for programming. CRS staff and implementing partner staff reported on the goals of THRIVE II and program outcome design.

#### Observations

In each of the six study communities, we conducted observations with 12 households. We conducted two observations per household for a total of 24 structured household observations. The research assistants and the community health volunteers (CHVs) worked together to identify households based on the following criteria: 1) a female caregiver participating in THRIVE II who had consented to observation, and 2) had an index child (6–24 months) as the primary focus. We received consent from mothers as they were identified as primary caregivers. If other caregivers (siblings, grandparents, fathers, etc.) were present or caring for the child, that information was included in the observation. An ‘index child’ was selected as the focus of observation as some households had more than one child between 6 and 24 months of age. Observations were conducted in Luo by research assistants who were residents of Homa Bay and Migori counties. Observations were conducted over two days by the same researcher in the same household; 4 h on day one, and 6 h on day two, to understand caregivers’ behaviors. Caregivers who participated in observations did not participate in FGDs. Caregivers were fully aware of being observed and were encouraged to continue with their activities as they would do in the absence of the observer. The use of two days of observations in the same household by the same observer was intended to minimize reactivity bias and to increase caregiver comfort in the presence of the observer. Half of the observations were conducted in households with an index child between 6-and 12 months, and half with an index child aged between 13-and 24 months. Research assistants used a structured observation tool to record food hygiene behaviors related to meal preparation, feeding, hygiene, sanitation, water collection, and handwashing. Research assistants also conducted household spot checks to assess the compound environmental sanitation and sanitation hardware (e.g. presence of handwashing station near food preparation area, presence of animal feces in food preparation areas, functionality and use of latrine). Observations were intended to give insights into IYCF and WASH behaviors that caregivers of CU2 practiced at home. Caregivers with children of different ages were targeted to enable observation of potential differences in hygiene behaviors.

Observations were conducted between 09:00 and 16:00 h; 09:00 was the earliest time that care group volunteers (lead mothers who spread basic health information to a maximum of 12 women or families in their communities) [49] would accompany research assistants to households. Caregivers usually granted permission for observations over their midday meal, enabling the research assistants a chance to observe their food preparation and hygiene practices. In the event that a caregiver expressed discomfort or refusal to be observed during food preparation, observers respected their decision.

### Data management and analysis

Focus group discussions (FGDs) were conducted in Luo, while key informant interviews (KIIs) were conducted in the language of the participant’s choosing- either Luo, Kiswahili or English, and audio-recorded. The FGD and KII audio files were uploaded to a cloud-based server, de-identified, transcribed verbatim in Luo and translated into English. Back translation of the transcripts was not done; however, transcripts were reviewed against corresponding audios by the field team managers to ensure the accuracy of translations. Detailed field notes from household observations were written in English and typed into Word documents. All individual files were password protected.

Data analysis began concurrently with data collection. The field team debriefed daily, discussing the emerging themes from the day’s data collection. Detailed daily briefing notes were maintained and shared with the research team via the cloud-based server. Thematic analysis [50, 51] was used to identify common barriers and facilitators to the targeted behaviors, including food preparation and hygiene, and developed these into a codebook. Through the use of the COM-B model (Table 1) of behavior change and behavior change wheel framework, deductive codes were developed and aligned to specific behaviors of interest and key behavior determinants – capability, opportunity, and motivation [47]. KII and FGD transcripts were then coded using MAXQDA v20.1.1. Four researchers met weekly to discuss iterations to the codebook and ensure that they had the same understanding and coded similarly. Ambiguous segments were discussed, and codes were adapted as needed. Observation data from the checklists were analyzed using Microsoft Excel, and observation notes were thematically analyzed, identifying common themes and patterns.

### Ethics

The research protocol was reviewed and approved by the Great Lakes University of Kisumu Research Ethics Committee (Kisumu, Kenya) (#GREC/1954/2017), the Government of Kenya National Commission for Science, Technology, and Innovation (Nairobi, Kenya) (NACOSTI/P/16/72200/13631), and Emory University’s Institutional Review Board (Atlanta, GA) (#IRB00090057). Research assistants read the informed consent to the participants in Luo. All participants provided written informed consent after it was read to them.

## Results

We collected qualitative data from 139 individuals, including mothers, fathers, and grandmothers (Table 2) along with observations data from 12 households. The responsibilities of caregivers—31% are housewives and 41% are engaged in business. The majority of participants collect water from outside the compound, with a greater percentage getting water from the lake. A number of participants do not have a latrine and use other places facilitating environmental pollution.

**Table 2:** Demographic data of FGD participants

Characteristic	Mothers	Fathers	Grandmothers
	Overall (*N* = 68)	Overall (*N* = 36)	Overall (*N* = 35)
Age in years (range)	28 (18–45)	38 (25–68)	56 (25–87)
Number of children	4	5	7
Age of oldest child	11	14	34
Age at birth of the oldest child	18	24	18
Number of people in the household	6	8	6
Number of people in the compound	9	8	10
Education, n (%)			
Completed Primary school	48 (71%)	17 (47%)	32 (91%)
Completed Secondary school	16 (22%)	14 (39%)	2 (6%)
Completed Tertiary school	4 (7%)	5 (14%)	1 (3%)
Occupation, n (%)			
Business	28 (41%)	7 (19%)	7 (20%)
Housewife	21 (31%)	0 (0%)	5 (14%)
Fishing	0 (0%)	5 (14%)	0 (0%)
Farmer	8 (12%)	13 (36%)	21 (60%)
Other	11(16%)	11(31%)	2(6%)
Latrine ownership, n (%)			
Yes	28 (41%)	23 (64%)	25 (71%)
No	40 (59%)	12 (36%)	10 (29%)
Sanitation access, n (%)			
Can access a latrine	43 (63%)	24 (67%)	25 (71%)
Cannot access a latrine	25 (37%)	12 (34%)	10 (29%)
Shares a latrine, n (%)			
Yes	46 (68%)	28 (78%)	27 (77%)
No	22 (32%)	8 (22%)	8 (23%)
Primary water source, n (%)			
River/lake/ pond/stream	31 (46%)	15 (42%)	18 (51%)
Piped water	18 (27%)	7 (19%)	7 (20%)
Water pan	6 (9%)	4 (11%)	4 (11%)
Deep borehole	9 (13%)	4 (11%)	1 (3%)
Open well	0 (0%)	4 (11%)	0 (0%)
Other	4 (5%)	2 (6%)	5 (15%)
Distance to a primary water source, n (%)			
Outside of compound	65 (96%)	35 (97%)	33 (94%)
In own compound	3 (4%)	1 (3%)	2 (6%)

**Fig. 1:**
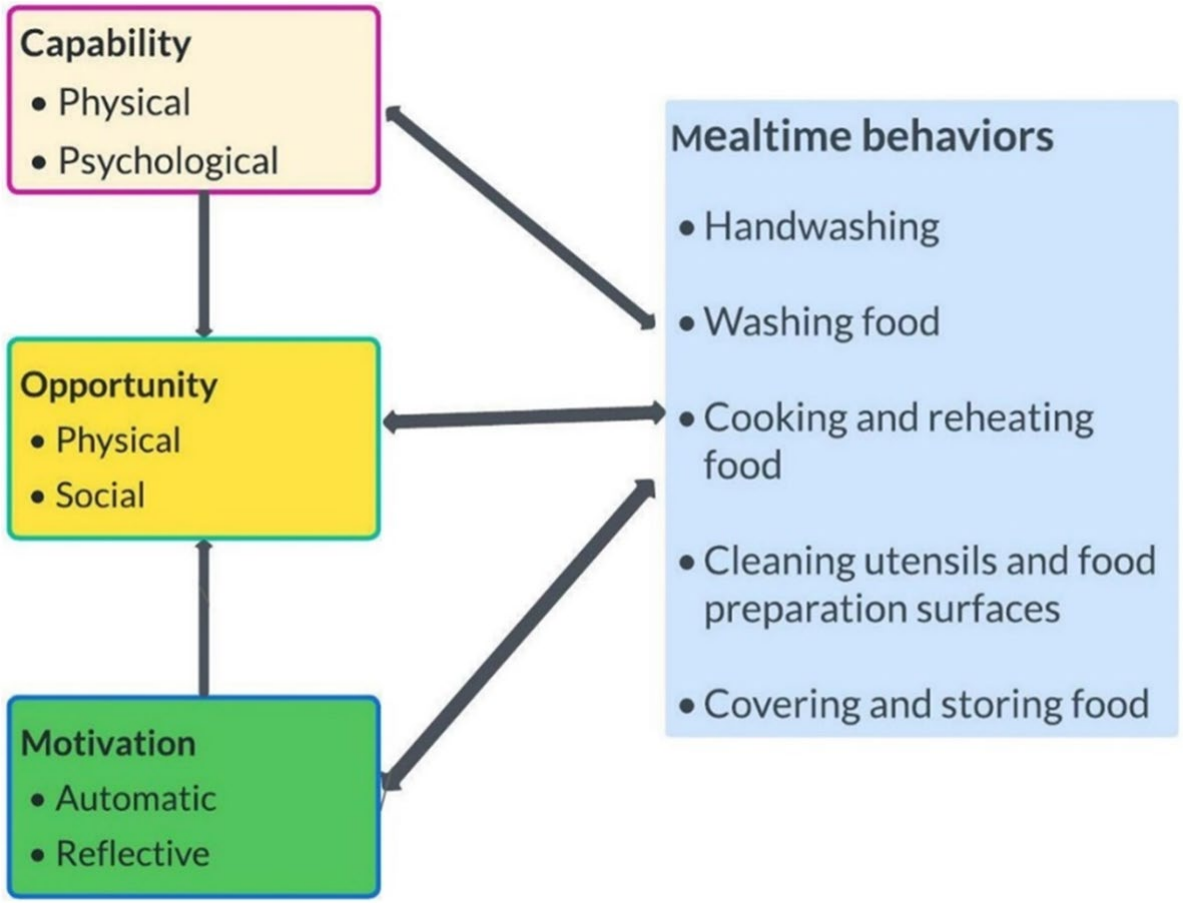
Capability, Opportunity, and Motivation interaction with focal mealtime behaviors [41, 52]

The findings on determinants of food preparation and hygiene practices are presented following the capability, opportunity, motivation, and behavior (COM-B) domains. Figure 1 is a summary of capability, opportunity, motivation, behaviors (COM-B) domains and their interaction with focal mealtime behaviors specific to food preparation and hygiene. The arrows indicate the potential influences between and within the domains and behaviors. We present results describing the barriers and facilitators to food preparation and hygiene practices organized by the COM-B domains as determined by the data from observations and discussions. We discuss the findings following this order 1) capability 2) opportunity and 3) motivation. We align the findings to focal mealtime behavior including 1) handwashing, 2) washing of food, 3) cooking and reheating food, 4) cleaning utensils and food preparation surfaces, and 5) covering and storing food.

### Capability

Physical capability was a facilitator of food preparation and hygiene practices (Table 1 for definition). Caregivers were observed to show physical capability in washing hands, utensils, and food; cooking food to safe temperatures; covering and storing food; and fetching water and firewood. However, psychological capability was both a facilitator and barrier to practicing food preparation and hygiene behaviors. Caregivers demonstrated knowledge and skills on handwashing, washing food, cooking food thoroughly, and covering food; some caregivers demonstrated a lack of knowledge and skills on critical times for handwashing, importance of reheating food, and possible food contamination due to prolonged storage period.

#### Handwashing

In interviews, caregivers reported psychological capability in the form of knowledge about how and when to perform the action (Table 1): they learned about handwashing from their school-going children, who taught them about handwashing from their school education. Grandmothers shared how they washed their hands “clean” using soap and air drying before serving food. Caregivers were observed at some handwashing events for themselves and their children: before food preparation, before eating or feeding the child, after eating, and post toileting (Tables 2 and 3). Mothers insisted that they were the ones to hand-feed their children since they knew best how to wash their own hands clean.

*“Here, I am the one who gives the child’s food when I want to feed her, it is me who has to feed her because I am the one who knows how I hand-wash. Now after washing my hands with soap and having dried is when I take food to feed my child.” Mother, Migori*

**Table 3:** Observed mother and grandmother handwashing practices and opportunities

Key event	Total opportunities *(N* = *103)*	*Washed with soap* *(N* = *3)*	*Washed with water* *(N* = *30)*	*Not washed* *(N* = *70)*
Before eating	14 (14%)	0 (0%)	8 (57%)	6 (43%)
Before feeding the infant/child	20 (19%)	0 (0%)	7 (35%)	13 (65%)
Before food preparation	16 (16%)	1 (6%)	3 (19%)	12 (75%)
After feeding the child	20 (19%)	0 (0%)	2 (10%)	18 (90%)
After eating	14 (14%)	0 (0%)	4 (29%)	10 (71%)
After cleaning baby post defecation	7 (7%)	2 (29%)	2 (29%)	3 (43%)
After adult toilet	7 (7%)	0 (0%)	3 (43%)	4 (57%)
Other	5 (5%)	0 (0%)	1 (20%)	4 (80%)

Although mothers and grandmothers reported knowing how to wash their hands, this was not reflected by observation data. Some caregivers did not wash their hands at critical times while others did not follow all the handwashing steps. Improved hand hygiene behaviors include 1) washing hands at critical times, 2) following all the handwashing steps, and 3) using soap and running water for handwashing [49]. Out of all the handwashing opportunities observed for mothers, mothers washing hands with soap occurred in only 1 out of 16 observation events before food preparation, accounting for 6% of observed events of food preparation, and 2 out of 7 (29%) of observed events after cleaning baby post defecation (Table 3).

Handwashing for children (Table 4) was done primarily by mothers and grandmothers, as they agreed that most children would not start washing their own hands until they were 4–6 years old. However, because children “touch dirty things,” caregivers noted that children’s hands needed to be washed more frequently.

*“That a small child, anytime they come from play and they want to eat, you have to wash their hands because where they walk, he/she doesn’t know even how to differentiate chicken feces, he/she will carry with her/his hands. So anytime you want to give something then you have to wash the hands clean with soap.” Grandmother, Homa Bay*

**Table 4:** Observed child handwashing by caretaker practice and opportunity

Key Event	Total Opportunities (*N* = 70)	*Washed with soap* *(N* = *0)*	*Washed with water(N* = *15)*	*Not washed* *(N* = *55)*
Before eating	20 (29%)	0 (0%)	9 (45%)	11 (55%)
After eating	20 (29%)	0 (0%)	5 (25%)	15 (75%)
After toileting	7 (10%)	0 (0%)	1 (14%)	6 (86%)
Other events (before drinking porridge, before breastfeeding)	23 (33%)	0 (0%)	0 (0%)	23 (100%)

Although the caregivers possessed the knowledge that children’s hands should be washed with soap, from observations, out of the 70 total handwashing events observed for children, none of the children’s hands were washed using soap (Table 4). This could be attributed to a lack of physical opportunity as discussed in further sections. Lack of psychological capability as a barrier was also noted with the grandmothers and mothers not following all the recommended handwashing steps. Grandmothers noted that they poured water into a basin, washed hands by dipping and without soap, poured out the used water, and rinsed hands with clean water. Caregivers explained that they would soak the child’s hands in water once, regardless of the hands being visibly or invisibly dirty..

“For a child who is less than two years, I don’t wash her hands the way I wash mine. I just soak it and then I remove it…because it doesn’t have germs”. Mother, Migori

#### Washing of food

Caregivers reported washing some foods but not others. Many fruits, including mangoes, bananas, guavas, sugarcane and oranges were not often washed. Some of these fruits like mangoes and guavas were eaten raw, not washed nor peeled. Grandmothers reported that sometimes when harvesting sweet potatoes or cassavas, they could not wait to go and clean them at home but would instead wipe out the soil, peel them using their mouth and eat them without washing. Caregivers reported eating fruits without washing when they were in places like markets or when traveling.

*“Sometimes I went somewhere and I am hungry, then I see a guava. I will pluck that guava and eat. But it is not supposed to be like that, I should pluck it and go with it to the house and wash it. And then I wash my hands well then I eat it. But because this normally happens to us, I walked to the market, I will buy a sugar cane and then I just start eating, and yet I had not washed that sugar cane.” Grandmother, Migori*

During observations, caregivers demonstrated psychological capability (Table 1) as a facilitator in washing food. Participants always washed “omena” (Lake Victoria sardines) before they were prepared; in interviews, participants confirmed food washing for sand and stone removal. In observations, tomatoes were washed approximately half of the time, and rice was carefully washed and sorted twice before cooking. Participants were observed to carefully wash sukuma wiki (kale) at home, instead of buying it precut from the market.

“I often buy kale before they are cut because when I go and wash, the sand does not come out so I buy kale which has not been cut and wash slowly, one by one, and then I slice…” Mother, Homa Bay

#### Cooking and reheating food

Caregivers were observed to cook their food thoroughly and during FGDs, they described how they would know that the food was well cooked. A typical meal that caregivers reported to prepare for both lunches and dinners was “ugali” (starch made from cornmeal, cassava, or millet flour), sukuma wiki (kale), and “omena” (Lake Victoria sardines). For children, porridge was a common food in the households. Caregivers describing how to cook porridge indicated that porridge is cooked once it boils, removed from the fire, and then returned to heat and left to boil.

*“You wash the cooking pot well and put it on fire. I pour water and then after pouring water, after it has boiled, I stir enough flour into a cup. After stirring and the water has boiled then I add to it, after I have stirred and it has become thick and I have added water, when it bubbles, I add sugar if I have one but when I don’t have sugar but I have lemon I can add the lemon. When it is cooked, I remove it.” Mother, Homa Bay*

Caregivers stated that they would warm leftover food before feeding it to the family. As observed, the index child’s (youngest child in the household age 6–24 months) porridge was stored in a thermos. Caregivers said that the thermos kept the porridge warm and caregivers would pour a small portion into a cup for feeding so that the rest does not spoil.

*“You become wise and tell her “Give me the thermos that has porridge so that I can feed it.” We should not give the baby cold porridge, we should feed it warm porridge and using only the cup not the spoon. Grandmother, Homa Bay*

#### Cleaning utensils and food preparation locations

Caregivers reported washing utensils used for feeding and food preparation on daily basis: physical and psychological capability were a facilitator to practicing the behavior. This task was usually the responsibility of the mother or another female family member and necessitated water and time. Many described a similar step-by-step process which included three rinses, the use of soap and an abrasive tool, and drying in the sun before storing in the house. However, from observations, only 4 out of 12 households had and utilized a drying rack.

*“When I wake up in the morning, I take utensils outside, I put water in three basins, one for cleaning and the other two are for rinsing, then I place – those that I have rinsed I allow to dry up, then I take them into the house, and cover them when they have dried, that’s how I clean my utensils.” Mother, Migori*

Cleaning infant cup covers with a nipple was a challenge. Many caregivers believed that baby bottles could be dirty and spread disease, relating this to the difficulty in properly cleaning the bottle including the nipple part. One grandmother described the difficulty of keeping a baby bottle clean.

*“…feeding the child milk or porridge using the baby bottle will expose it [the child] to infections...[t]here are times when it is washed outside and not washed on the inside and there might be some dirt remaining inside there. When the baby just feeds on it, he/she starts having diarrhea.” Grandmother, Homa Bay*

Although maintaining the cleanliness of bottles and nipples was a concern cited by caregivers, sippy cups and bottles with nipples were observed being used to feed porridge to younger children.

#### Covering and storing food

Caregivers exhibited a lack of knowledge about the risks of food contamination when cooked in large quantities and stored for long periods. Some of the caregivers stored food for more than four hours before reheating and/or eating again, usually during the day when the caregivers were away for work. Left-over food was also stored to be eaten the next day. Food was covered due to concerns about food contamination from household animals and flies; however, caregivers believed that food needed to cool before covering to prevent it from spoiling.

### Opportunity

Physical and social opportunity influenced caregivers’ behaviors related to food preparation and hygiene practices (see Table 1 for definitions).

#### Handwashing

Basin, water, and soap were the most frequently mentioned handwashing materials caregivers possessed. Caregivers mentioned having a jerrycan (a container made of plastic, that has a narrow mouth and is used for holding water) with a hole hung near the latrine and filled with water for handwashing after latrine use. Observations showed that none of the caregivers had a designated place for handwashing near the cooking area. Bar soap and laundry soap were generally available in small shops and markets; however, caregivers lacked the physical opportunity to practice handwashing with soap. While several caregivers reported soap as inexpensive, others reported the cost of soap as a barrier, thus the absence of soap for handwashing.

*“You can fail to get soap if you do not have money to buy it with, and you do not have any place that you can get it from, and so you will just wash your hands with just water which does not remove germs.” Grandmother, Homa Bay*

More than half the caregivers collected water from sources outside their compounds (Table 2). Water was closer in communities near Lake Victoria than in hilly areas, where women reported fetching water could take more than two hours. Caregivers who lived far from the water sources often minimized water wastage and prioritized water for other needs other than handwashing.

“We are far from the lake, we are at the furthest end, but we collect water from the lake.” Grandmother, Homa Bay

Caregivers prioritized purchasing soap for washing clothes, dishes, and bathing over handwashing. At times people adapted to a lack of handwashing with soap and used other low- or no-cost materials for handwashing including ash or water alone.

#### Cooking and reheating food

Physical opportunity was a facilitator and a barrier to cooking and reheating food. Cooking and reheating of food were facilitated by the availability of water, firewood, and time. Collecting water and firewood, typically women’s responsibilities would be prioritized over other household chores if they were not accessible near the households. Sometimes women bought firewood; this influenced how often mothers prepared and reheated food, but did not hinder cooking and reheating food. Food was cooked in large quantities and stored to save on cooking fuel, time for collecting fuel, and time for cooking.

In Homa Bay, lack of physical opportunity like water, money, and cooking fuel was a barrier to practicing food preparation and hygiene practices. Per government policy, the Ministry of Environment and Forestry guarded the hills nearby to prevent firewood collection. Food affordability limited which foods were purchased and eaten; these usually included kales, “omena” (Lake Victoria sardines), and “ugali” (starch made from cornmeal). In terms of washing food, participants stated that when they were in the market, on a journey, or away from home, they would eat some foods like fruits without washing because of a lack of water to wash them. High ambient temperatures meant that caregivers did not always cover some foods (meats, fish, “githeri” (a mixture of maize and beans), vegetables including kale, legumes including beans, and green grams (lentils) after preparation or when storing. Caregivers said that if these foods were covered while hot, moisture would accumulate in the storage pot which would make the food spoil faster. In observations, only four households out of 12 had food covered at the time of the visit.

*“I also fry kale after cooking everything…I take it and go cover, or hang it on a rope or put it in the cupboard. Before you will reach lunch, you are forced to reheat it because you fried it with onions…If you cover it, it will go bad.” Mother, Migori*

Typically, most caregivers reported preparing food three times a day as a norm, but observations and statements showed this was not always the case. A mother stated, “*In this house, I do my day’s food preparation- that’s breakfast and lunch all at once.”* Families also shared responsibilities; co-wives cooked together, both male and female teenagers cooked and practiced household chores. Mothers-in-law cooked for their daughters-in-law post-childbirth. Participants placed value on food preparation hygiene when participants used the basin designated for washing utensils as opposed to the basin designated for bathing. However, the reasoning behind it was not mentioned.

*“How I prepare this green banana, I put it in a trough, I take another trough or a clean cooking pot, not bathing trough, it is a utensils trough that you can even pack your food on which you had prepared, then you put it down with water...” Mother, Migori*

Cultural norms and religion influenced whether caregivers reheated food. Often elders preferred foods like “githeri” (mixture of boiled maize and beans), boiled sweet potatoes, and boiled maize, to be eaten when cold. The Seventh-day Adventists and “Roho” faithful followed a church doctrine preventing them from lighting a fire on the Sabbath. They prepared meals on Friday evening to last through Saturday evening.

Resource sharing by caregivers was discussed at length: one participant mentioned that she could be given money by neighbors and would use that money to buy soap for cleaning utensils. Some caregivers also had savings and loan groups, where they contributed money, loaned out to members, and paid back with interest within a time period, and merry-go rounds where money is contributed and given to one person. Caregivers used the money received for household needs, including purchasing food. Some fathers bought food or provided other caregivers with food money.

#### Cleaning utensils and food preparation locations

Physical opportunity- water, soap, time, available designated places for cleaning and drying-facilitated washing of utensils. Utensils were cleaned at least once: 1) in the morning, 2) before cooking, 3) immediately after cooking (during the day), or 4) at night after cooking. The timing was dependent on caregivers’ engagement in other chores. Utensils were cleaned in designated places either outside the house but within the compound, in the kitchen, or at the lake. Utensils were dried on a rack, plastic crates, portable trolleys, basins, or hanging on a rope or the fence. Alternatively, caregivers hand-dried utensils to prevent wind disturbance or animal contamination.

*“When I go to the lake with the utensils, I carry three basins, one big basin which carries all of them, two other basins where I am going to wash them in… I first put cups, wash and rinse then put them in the big basin then I wash the plates and dishes and put them there then I get to the cooking pots, scrub, and after scrubbing, I will arrange them all in the big basin and bring them home. I have a crate at home and a table in my kitchen, so after coming with them from the lake, I arrange in that crate in the kitchen.” Mother, Homa Bay*

Some participants had separate soap for cleaning cooking pots which they called “black soap”, noting that this soap made the cooking pots sparkle and shine.

The presence of the kitchen as the most common food preparation space was a facilitator to optimal food preparation and hygiene as it provided space for varied food preparation practices. Kitchens were used for varied purposes including food preparation, cooking, utensil cleaning, storage space, and as a sleeping area for children and animals, including chickens. Other food preparation locations were rarely mentioned. Sweeping the kitchen floor was a norm as reported by caregivers, and was conducted daily primarily to remove chicken and other animal droppings.

#### Covering and storing food

Physical opportunity of having different methods of food storage was a facilitator to optimal food storage behaviors. Caregivers used metallic cooking pots, plastic jugs or cups, metallic hot pots, and thermos flasks. Observations showed that caregivers often stored food inside the house, sometimes up on top of a cupboard. Other caregivers hung their food from a rope designed to hold items tied mostly to the roof, to protect it from dogs, cats, chickens, and children.

### Motivation

Reflective and automatic motivation influenced food preparation and hygiene practices among caregivers (see Table 1 for definition). Reflective motivation was seen when caregivers evaluated the process of thoroughly cooking food, and this included prioritizing water for washing food before it was cooked or eaten. Subsequently, caregivers expressed automatic motivation through disgust related to flies landing on food and eating germs.

#### Handwashing

Automatic motivation (Table 1) facilitated and hindered food preparation and hygiene practices. The presence of visible dirt on some caregivers and children’s hands was a visual cue for handwashing since they did not want to ingest germs. However, some caregivers believed that hands were only dirty if dirt was visible. This belief affected the frequency of caregiver handwashing and children’s handwashing by caregivers even though they could have been microbiologically contaminated.

*“This [handwashing with soap] will be of benefit to the child because now it [child] is healthy, the child’s health is good. Diseases that come in like diarrhea that attack the child abruptly will not be there because we are taking care of the child well, it [child] does not eat germs, it does not eat dirt.” Grandmother, Migori*

Caregivers reported that handwashing with soap would prevent them and the children from “eating germs” thereby preventing children from getting illnesses like “diarrhea and cholera”, considering the value of handwashing and risks evaded by the practice. However, some caregivers prioritized water for alternative uses over water for handwashing.

#### Cooking and reheating food

Caregivers expressed reflective motivation when they explained the porridge preparing procedure. Caregivers indicated that for porridge to be cooked, it must go through the following steps: 1) placed over a fire until it boils, 2) removed from the fire to prevent overflowing, 3) returned to the fire, and 4) left on the fire to boil “until the foam clears”. These practices indicated a thoughtful step-by-step evaluation process from preparation time to when the porridge is well-cooked ensuring properly cooking food.

*“When I want to prepare porridge, I light up the fire, take enough water that I will use to prepare it. I take a jug, I take enough flour and pour it there, I stir well and pour it. When it is maize flour after pouring the water, I stir well until it is well mixed, and when it is boiling and almost ready and it bubbles up, I add water, I add sugar if I have, and if I also have lemon I add and I leave it to boil because maize flour must boil well until all the bubbles disappear and it is then that I remove it and pour inside a big jug.” Mother, Homa Bay*

Although caregivers shared their step-by-step plan to thoroughly cook food, due to other competing demands, caregivers prioritized other tasks compared to reheating or cooking fresh foods for children.

#### Cleaning utensils and food preparation locations

Reflective motivation was a facilitator to cleaning utensils and food preparation locations. Caregivers had a specific routine for cleaning utensils which included a thoughtful evaluation of what and how cleaning took place. Caregivers had designated times for washing utensils and a clear process included scrubbing, three rinses, and drying under the sun before the utensils are used.

*“How I clean my utensils, I wake up in the morning then buy water from the vendor. I have specific soap for utensils kept in a tin, I take that out together with my utensils. First, I rinse the food remains from them [utensils], then place them into three categories, in three troughs. First, I soak and rinse to remove dirty water, then place in clean water, then use soap on the sponge to scrub as I place in the next basin then I rinse on another basin, then place in another basin. After I have finished and scrubbed the cooking pots, I take a clean towel and wipe them all, rearrange them in the trolley, then take them inside the house.” Mother, Migori*

Due to other competing priorities, caregivers prioritized other tasks over optimal food hygiene. For instance, based on caregivers’ conscious decisions, high priority was placed on spending time going to work compared to spending time cleaning utensils.

Automatic motivation was a driver to cleaning utensils: disgust drove most actions. One participant was keen about leaving dirty utensils outside the house, noting that they would attract flies from the latrine which would land on the utensils, a sight she never wanted to see. Some caregivers mentioned that they would wipe their utensils with designated towels before storing them. These towels would be washed to avoid attracting “small flies” from landing on the towels, a sight expressed as disgusting by participants.

*“When I want to clean utensils, I do that in the house … when I was young, I was taught that when cleaning utensils outside where latrine in nearby, and you leave them unwashed outside as you still rearrange your house, latrine flies can land on the utensils…” Mother, Migori*

#### Covering and storing food

Caregivers also expressed disgust with flies landing on uncovered food thus motivating them to cover their food to avoid such instances. However, some caregivers consciously avoided covering food, citing that moisture from hot food when covered, would make the food go bad. This provided an opportunity for possible contamination by flies and other items which could get into the food.

## Discussion

This study explored facilitators and barriers to practicing optimal food preparation and hygiene behaviors (Table 5) in Homa Bay and Migori Counties in western Kenya. The behaviors of interest on focus were 1) handwashing, 2) washing food, 3) cooking and reheating food, 4) cleaning utensils and protecting food preparation spaces, and 5) covering and storing food. The identified facilitators and barriers were categorized by the COM-B domain to better understand the relationships between the determinants; our ultimate objective was to develop and test a theory-informed intervention [41, 42]. Several critical barriers emerged from our data especially related to physical opportunity including resources and facilities and social opportunity such as sociocultural norms. These barriers, compounded with poverty and high HIV prevalence within the study population could have a major implication on hygienic food preparation practices. While many caregivers demonstrated knowledge and skills, had resources/facilities, and were motivated to practice most food preparation and hygiene practices, others lacked knowledge and skills (capability) to practice certain food preparation and hygiene behaviors, namely, prolonged food storage in an unsafe environment. Many caregivers lacked resources (opportunity) like soap, water, firewood, and time to practice safe food preparation and hygiene practices. Socio-cultural norms related to handwashing, washing, reheating, and covering food also hindered optimal food preparation and hygiene behaviors. Leveraging existing facilitators (knowledge and available resources) to address specific barriers to practicing food hygiene and preparation behaviors can increase people’s motivation to enact specific behavior through encouraging continuity of positive practices. Experiences and recommendations from previous intervention programs aligned with our findings [10, 11, 53]. Based on this formative work, we recommend that programs that aim to address food preparation and hygiene practices (Table 5) utilize theory informed approaches to inform their interventions, considering the context in which the interventions are implemented. This study highlighted how determinants to food preparation and hygiene behaviors were influenced by capability, opportunity and motivation and how these domains had an influence in part or whole to achieving the optimal food preparation and hygiene behaviors in this setting.

**Table 5:** Definitions of optimal food preparation and hygiene behaviors [41, 52]

**Behavior of interest**	**Definition**
Handwashing	Handwashing with soap and water by the caregiver and the child at critical times (before eating; before feeding child; before, during and after food preparation; after defecation of caregiver and/or child; after cleaning area/tools for child defecation)
Washing food	Washing of fruits and vegetables before eating
Cooking and reheating food	Cooking of food until boiling/very hot; reheating left-over food to appropriate temperature (until very hot) before eating or feeding the child
Cleaning utensils and food preparation surfaces	Washing and drying of utensils and food preparation surfaces after use Food preparation space cleanable, inaccessible to animals and elevated off the floor Utensils stored dry, free of dirt and debris, and inaccessible to animals and children
Covering and storing food	Food covered after it is removed from fire. Food inaccessible to animals, children, and flies. Storing food at ambient temperature for no more than 4 h

### Capability

A minority of caregivers lacked knowledge on the critical times for handwashing, skills on washing food, drying utensils, covering food, and storage of food, similar to studies in rural Malawi and Nepal [10, 11, 54]. Children’s hands were not washed as often as adults’ hands and in most cases, soap was not used. Previous interventions have targeted the promotion of food hygiene behaviors through different information, education, and communication (IEC) strategies. Intervention implementation on food hygiene in Malawi and Vietnam used IEC strategies like demonstrations, games, rewards, songs, workshops, newsletters, loudspeaker announcements, and flip chart communication to create awareness and educate people on food hygiene practices [37, 55]. In Pradesh India, Tidwell et al. found that targeted messages through television commercials or messages delivered via mobile phones produced a significant increase in handwashing behavior among mothers [56]. Although some interventions already exist that focus on measures to increase knowledge and skills on food hygiene practices, these interventions may need to be more targeted. For instance, interventions that address handwashing at critical times may need to lay more emphasis on the importance of training children on handwashing at critical times, even if the hands are not visibly dirty, to instill the habitual practice as they grow. Additionally, visual forms of education which portray the presence of germs on people’s hands could be used to facilitate peoples’ understanding on reality of invisible germs.

### Opportunity

Practicing behaviors requires an enabling physical environment: resources, time, and social norms (physical and social opportunity), and these were both enablers and barriers to practicing food hygiene behaviors. Evidence suggests that barriers to physical resources negatively impact food hygiene behaviors [12, 39]. Data from this study revealed that some caregivers lacked materials, this aligned with literature that reported a similar lack of critical materials (soap, water, firewood, time, handwashing station near food preparation area, and cues to trigger action) that contribute to not practicing food preparation and hygiene behaviors [11, 39, 40]. In food hygiene studies in Nepal and India, participants in FGDs reported practicing handwashing using soap, but observations revealed a lack of use of soap for handwashing [10, 57]. Although disconnect exists on practices reported to practices observed, handwashing is a common practice, and is an indication that handwashing with soap may be a particularly acceptable and feasible practice to target through targeted handwashing messaging [35, 58]. In Kenya, a study reported a substantial increase in the presence of soap and water together within 10 m of the food preparation area after intervention implementation [42]. Changes to physical and social opportunity have been addressed in interventions by the WASH Benefits study and the Safe Start trial. In these intervention trials, caregivers received handwashing stations, soap, food storage containers; and education and motivational messaging thus demonstrating improved knowledge and practice on proper food hygiene practices [58, 59]. Although the WASH Benefits trial reported insignificant reduction in diarrhea after intervention implementation in Kenya, the researchers also noted inconsistent promoter support [58]. Support from community health workers through existing groups like neighbor groups within the care group model have demonstrated adoption of food hygiene and preparation practices [42]. Education combined with provision of infrastructure in addition to consistent and continued CHV support, may facilitate hygienic food preparation practices.

The availability of infrastructure is a strong indicator of the successful performance of desired targeted behavior [37, 39, 60]. In Kenya, the Ministry of Public Health and Sanitation has regulations on drying racks, and as part of THRIVE II, an emphasis on food hygiene practices had been placed on washing and drying utensils using a drying rack placed in the sun, however, many households in our study communities lacked one. Caregivers had developed innovative approaches, which included the utilization of alternatives (crates, buckets) that facilitated the drying of utensils, a clear indication that solutions to some of these challenges exist within communities and recognizing such is key. Programs that implement interventions related to food hygiene in communities may need to identify existing innovative approaches within these communities and integrate them into their planned interventions implementation.

Social cues and cultural norms (social opportunity) were major barriers to food preparation and hygiene practices [61]. Food preparation in large quantities was a common practice due to lack of time, competing demands, and cultural norms. Prioritization of tasks to address time and work demands could be achieved through task-sharing among household members. In South Africa and Kenya, studies incorporated fathers and grandmothers in maternal and child nutrition practices and reported the contribution of fathers in decision making, in addition to expressing a preference for being more involved in maternal and child nutrition and care practices; and grandmothers providing childcare, nutritious food, and social support to mothers [62, 63]. Interventions could employ such strategies in communities where women bear the most burden of working and taking care of children like in communities in our context, to relieve them of some burden, and focus on food hygiene and preparation practices.

In our study areas, foods were stored uncovered due to the belief that they would spoil, and this could also be dependent on knowledge of food spoilage [42]. Some of the barriers to food storage could be addressed through the use of current technology for instance refrigeration. However, in this context, other factors like lack of electricity and affordability (cost) can hinder embracing technology, making it more challenging to address such beliefs. Some interventions have employed both interpersonal counseling and a community-based approach to sensitize communities with targeted messaging [42, 55]. Within the context of this study, sensitization of community educators and religious leaders and further engagement of community health volunteers to facilitate behavior change messaging could be employed.

In our study communities, women were involved in household responsibilities that required resources (physical opportunity), but they lacked monetary decision-making powers that would enable them to acquire resources, and this influenced their ability to prioritize tasks. Food hygiene interventions that have utilized a whole household-based approach have been effective at addressing barriers to food hygiene and preparation practices [53]. In particular, in Burkina Faso, gender-inclusive approaches reduced the workload and increased monetary decision-making power for women [64]. Labor-saving technologies that allow for utilization by both men and women, for instance, improving household water sources, use of alternative fuels or improved stoves, and awareness-raising for both men and women on hygiene could increase gender inclusivity [65]. Care for children, especially feeding is viewed as a collective responsibility to mothers, grandmothers, siblings, and sometimes neighbors, a finding that has been reported in other low-income settings [40, 66]. In western Kenya, interventions that targeted male involvement with decision-making on complementary feeding, and grandmothers’ involvement in the provision of positive social support improved some infant feeding practices [53, 63, 67]. Additionally, relieving women from taking up all the household responsibilities through task shifting, role sharing, training other household members including fathers, siblings, and grandmothers may help in improving food hygiene practices in the household by allowing time to prioritize proper food hygiene practices.

### Motivation

Motivation, as observed from our data, was driven by prioritization, specific routines and plans, disgust, and beliefs, and both facilitated and hindered practicing food preparation and hygiene behaviors. Motivation to enact behavior can be limited by capability and opportunity, as these two domains are prerequisites to motivation. Disgust related to visible dirt and flies facilitated handwashing, covering of food, and washing of utensils similar to reports from studies elsewhere [39, 68, 69]. Belief that dirty hands could carry microbes that can make someone sick facilitated handwashing, although the absence of visible dirt hindered the practice. Since motivation always involves a competition between alternative behaviors, getting people to enact behavior may involve both decreasing and increasing motivation to enact behavior [47]. In this context, the importance placed on specific practice influenced prioritization, which was also influenced by physical opportunity; for instance, distance to a water source informed how water was utilized in the household.

Our data informed a WASH and nutrition intervention grounded in the COM-B theory of behavior change. The findings were used to develop a strategy that added minimal inputs to enable behavioral change, including information, education, and communication materials (pledge cards and food hygiene cards), and hardware (washbasin, pitcher, and soap for handwashing stations and mesh food covers) [42]. The intervention implementation based on this formative research resulted in improvement in hygienic food preparation outcomes and handwashing practices [42]. Interventions based on culturally acceptable, locally available, and low-cost interventions have increased handwashing practices among women after involving community health workers in providing hygiene messages to mothers through household visits [55, 70].

## Strengths and limitations

A strength of this study was the ability to triangulate across interviews, focus group discussions, and observations. This approach helped verify practices and ensured that our findings were grounded in participants’ experiences. The two-day observations in the same households helped reduce the observer’s influence on participants’ behaviors since the participants were accustomed to the observers’ presence and may have returned to their natural interaction by the second day. However, the timing for our observations (determined by the families and community health volunteers) limited us in observing major cooking events and related food hygiene practices which often happened earlier than 9 am, and later than 4 pm. Primary caretakers were also occupied by other responsibilities which led to them leaving the CU2 under the care of someone else while attending to those responsibilities. This narrowed the focus of the observation as caregiver-related behaviors would be minimal. Although we were able to get rich data from the FGDs with an understanding of caregivers’ behaviors, attitudes, and perceptions that determined food hygiene and preparation practices, some subjectivity, and researcher bias could have been experienced. However, these were minimized through interviewers practicing reflexivity. Additionally, all the food-related activities did not undergo any laboratory tests to quantify microbial risk and our results were based on observations and interviews alone. The understanding of the determinants to food preparation and hygiene practices could inform future formative research on WASH to explore the important domains aimed at addressing specific challenges in different settings.

## Conclusion

This study emphasizes the importance of understanding the existing facilitators and barriers to behavior change interventions for food hygiene, to guide interventions in building on existing community strengths in capabilities, opportunities and motivations, and addressing gaps. The understanding of individual knowledge alone is insufficient, as individual behavior is determined by a host of interacting personal, structural and social constructs, and utilization of COM B model provides a broader understanding of these determinants. Limited knowledge and lack of availability to see the presence of pathogens, decreases the importance of practicing certain behaviors (capability), including handwashing with soap (especially the child’s hands) and reheating and covering food, influence the motivation to practice those behaviors. Motivation to practice certain behaviors was also impacted by social opportunity including time, work demands [7], and social norms. The lack of water due to distance to a water source, firewood, lack of infrastructure to do washing in the kitchen, and sometimes money (opportunity) also affected motivation to practice certain behaviors. Addressing the myriad challenges to optimal food preparation and hygiene practices requires an integrated intervention that leverages the positive drivers that are locally available, accessible, and relatable to the communities [42]. The use of COM-B domains – capability, opportunity, and motivation – further highlighted the relationship between the domains with a clear indication of how one domain influenced the achievement of practice with the other domain. Categorizing these determinants using the predetermined COM-B behavior change theory supports the development of a theory-informed intervention [7].

## Supplementary Information

**Additional file 1.**

**Additional file 2.**

## Data Availability

The data analyzed during this study are not publicly available as all data are confidential but are available from the corresponding author on reasonable request.

## Abbreviations

COM-B: Capability, opportunity, motivation, and behavior
WASH: Water, sanitation, hygiene
HACCP: Hazard Analysis Critical Control Point
RANAS: Risk, Attitude, Norms, Ability, and Self-Regulation
BCD: Behavior Centered Design
CU2: Children under 2 CRS- Catholic Relief Services
HIV: Human Immunodeficiency Virus
AIDS: Acquired Immune Deficiency Syndrome
FGD: Focus group discussion
KII: Key informant interview
CHV: Community health volunteer
IYCF: Infant young child feeding
IEC: Information, Education, and Communication
IRB: Institutional Review Board
GREC: Great Lakes Research Ethics Committee
NACOSTI: National Commission for Science, Technology, and Innovation
ECD: Early Child Development
HHCDO: Homa Hills Community Development Organization
MOSGUP: Mercy Orphans Support Group
