# Supplementary material for: Determinants of food preparation and hygiene practices among caregivers of children under two in Western Kenya: a formative research study
Source: BMC Public Health. 2022 Oct 6;22:1865. doi: 10.1186/s12889-022-14259-6 (PMC9535979; doi:10.1186/s12889-022-14259-6)
Supplement: Supplementary file 1 — Additional file 1. [file 12889_2022_14259_MOESM1_ESM.docx]

**Additional file 1**

**FGD for Mothers WASH**

**Toilet Use**

Objectives: Describe societal/cultural, environmental, family, and individual drivers/barriers of:

- The presence and use of household toilets and places to wash hands.
  - Domains: social norms; availability; cost/accessibility; decision making; knowledge; ability

**Child Feces Disposal**

Objectives: Describe societal/cultural, environmental, family, and individual drivers/barriers of:

- Disposal behaviors of infant and young child feces.
  - Domains: social norms; shared cultural beliefs; availability of toilet; availability of potty/training mats/trowels, etc.; decision making; knowledge; ability;

**Hygienic Play Areas**

Objectives: Describe societal/cultural, environmental, family, and individual drivers/barriers of:

- hygienic play behaviors (creating a protected, hygienic play environment for the infant; deter contact between animals and young children; ensure children don’t play in areas frequented by animals.
  - Domains: social norms; availability; cost/accessibility; resource (raw materials) availability; support; decision making; knowledge; ability; perceived negative consequences

**Deworming**

Objectives: Describe societal/cultural, environmental, family, and individual drivers/barriers of:

- deworming behaviors for children >1 year, women of reproductive age, and pregnant and lactating women.
  - Domains: availability; accessibility; knowledge; agency; personal beliefs/trust;

**Notes:**

- Exact wording of questions will change based on feedback from local research assistants.
- Prior to FGD, participants will be read the consent form and will be asked if they would like to voluntarily participate. Participants will be provided with numbers and must fill out demographic forms once they have consented. Full details to be found in the SOP

| Setting Ground Rules |
| --- |
| **Facilitator Notes:**  The facilitator should discuss ground rules with the participants. Facilitators should brief participants to:   - Put their phones on silent. If a participant needs to answer the phone, they should quietly leave the room. - Take care of their consents (place out of the way) - Say their number before sharing their thoughts and opinion - Speak one at a time - Be aware that their participation is voluntary. If they need/choose to leave at any point, that is allowed. - Be aware that there is no right or wrong answer. This is a participatory exercise and all are expected to participate. - Protect confidentiality. All responses shared in the FGD should not be shared with anyone outside of the group.   **Facilitator Notes:**  The facilitator can ask people to contribute their own ground rules to the group as well. Ask if people have any questions before beginning. |
| Opening Questions *Opening questions are intended to build rapport and gradually lead into primary/key questions* |
| Opening Questions: Introductions |
| Each team should come up with a basic icebreaker or some closed questions that each person in the group can answer. ***Facilitator Script:*** Let’s go around and have each participant tell us   1. How many children do they have? 2. What they might do on a typical day? (Make sure to cover this thoroughly focusing on women’s activities related to water, sanitation, and hygiene) 3. Facilitators can use other icebreakers or conversation starters as appropriate |

**WATER, SANITATION, AND HYGIENE**

**Activity: Discussion of Sanitation Facilities**

| Defecation Locations | |
| --- | --- |
| Where do_____________typically urinate?   - - Men   - Women | - Women   - Pregnant   - After giving birth   - Lactating   - Menstruating |
| Where do ____________typically defecate?   - Men (old, young, boy) - Women (young unmarried, married, old women) | - Seasonal? - Time of day? - Women (pregnant, after giving birth, lactating, menstruating) |
| Where do children in the community usually urinate? | - Young child (infant) - Child close to 2 years? |
| Why do people prefer toilets to urinate and defecate? Not prefer? | - **Benefits?** - **Negative Consequences**-what might happen if someone is not urinating and defecating in the toilet? Consequences for   - Individual   - Household   - Community - **Time**. Different preferences for toilet or open defecation depending on the time - **Place.** Home/out and about - **Sharing.** Are there people who might not share toilets with each other? People in a family who would use a toilet and people who would not? |
| What would people say if they knew someone was defecating in the open? |  |
| **Child Feces Disposal** | |
| Where are all the places a CU2 might defecate? | **Notetaker instructions:** Make a list   - Is there anywhere else you can think of? |
| What places are   - most common? - Least common? - Best? - Safest? | - Why? |
| What influences where a child defecates? | - Age? - **Access** to the latrine - **Access to potty**, supporting tools - **Care of child**- Who is watching the child? When? Different in the day? Night? - **Time of day?** - **Convenience** Locations that are easier to clean? Which are easier/harder to clean? - **Location**: Home/away? - **Safety** |
| When are children old enough to use a toilet? | - Who teaches children? When? - Barriers? |
| If a family has access to a toilet, are there times when the child will not use the toilet? | - Why? |
| **Feces Disposal and Cleansing** | |
| What are some reasons that you don’t dispose of a childs’ feces in the toilet? | - Time - Cue to Action. Where is it normally put? - Where is it commonly disposed of? - Left in open. Why? How do you feel about this? - Why might people who have a toilet not dispose of children’s feces there? |
| What do you think is the safest way to dispose of child feces? |  |
| When do disposal practices change over time? | - Season - Time of day - Age of child (ability to walk) - Diarrhea |
| What are typical ways that child feces is transported? | - Clothes - Hands - Leaves - Tools - potties |
| Who usually disposes of children’s feces in a household? | - Who else might help? - How might the person impact the disposal method? |
| What are different ways to clean a child’s bottom after they defecate? | - Different materials? - Most common? - What do you think is safest? |
| Where might the water that is used to wash soiled (from poop) clothes, rags, or potties be disposed of? |  |
| What about cloth or rags, what happens to these? | - Washed/reused - Disposal |
| Sanitation facilities-Resource Allocation |  |
| What motivates a family in this community to have/build a toilet? | - Barriers to toilet ownership - Physical Environment - Sustainability - Available hardware? - Affordable? - Decision-making-who decides whether to spend money to get a toilet? - Messaging |
| Do some people in this community share toilets? | - How many people? Who shares toilets? Who does not share the toilet? |
| In your community, what are toilets usually made of? | - Why? Barriers to toilet building? - Hardware? - Maintenance? Time? Knowledge? |
| Who is normally responsible for cleaning toilets? | - Why? - Does this change when more people are using it? |

**Maternal and child handwashing**

| Can you describe how mothers typically wash their hands? |  |
| --- | --- |
| What about young children’s hands, are they washed similarly as adults? Why or why not? | - Babies - Children under 2 - Who washes children’s hands? - **Age** At what age do children wash their own hands? |
| **Instructions for Facilitators:** As participants answer the next questions, in their notes, the notetaker makes a chart on a sheet of paper that lists the mother in one column, the baby in the second column, the times before activities in the third column, and the time after activities in the fourth column. The facilitator should prompt in the following order: | |
| Can you tell me about all the events that occur on a day when it is necessary for mothers of CU2 to wash hands? | - **“**A mother will wash her hands *before* she _____________?” Why? - **“**A mother will wash her hands *after* she _____________?” Why |
| When are important times for mothers to use soap when handwashing? | - Are there certain events where a mother would use soap? Why? |
| When are times when it is necessary for CU2 to wash hands? | - **“**A mother will wash her childs’ hands *before* she _____________?” Why? - **“**A mother will wash her childs’ hands *after* she _____________?” Why - **Probe for**   - Eating (mother)   - Feeding a child   - Preparing food   - Farming   - After coming in contact with animals   - Defecating   - Washing a child’s bottom after defecation |
| When is it a good time for a mother to wash her____________ ‘s hands with soap   - Baby - CU2 | - Why? - Potential benefit? - Potential harm   - Self   - Others   - HH   - Community? |
| After the participants have listed important times for mothers and children under 2 to wash hands, The notetaker should put up the chart for mothers so they can see any differences. The facilitator should compare the two columns and probe on differences between the times mothers and babies wash hands and when they might use soap during a handwashing event. | |
| What are barriers to hand-washing? | - **Time.** Self, child. - **Affordability.** Soap - **Accessible.** Where does the water in your community come from? - **Theft.** Soap, hardware - **Maintenance**. Water, station, etc. |
| Where do mothers wash their hands? Can you please describe the places/hardware? | - **Different places** - **Hardware/facilities.** Basin, infrastructure, lake, tippy tap. - Near toilet? - Who else uses it? |
| Where does the water come from? | - **Primary Source?** - **Accessibility/Distance.** Where is the water source located? - **Alternative sources of water.** Seasonality - **Responsible?** - **How often replaced?** |
| Where does the soap come from? | - **Type**. What kind of soaps do people use? - **How often replaced** - **Affordable** - **Decision making.** Who decides whether to spend money on the soap? Why? |
| How common is it for toilets in this community to have water and soap near them? | - **Why/why not?** - **Barriers** |
| **Household Water Treatment** | |
| You said previously, that people got water for handwashing from___________(refer back to the previous section.) What is the primary source of people’s drinking water? | - **Accessibility/distance** Where in relation to people’s homes? - **Alternative sources of water.** Seasonality. Different sources for different purposes. - **Responsible.** Who gets water? - **How often?** |
| What do people do to make their water safe for drinking? | - **Treatment.** Bleach, Pur, water guard - **Boil.** If boil, how do you boil the water to make it safe? How do you know when it is safe? - **Filter.** - **Rainwater** |
| What makes it difficult for people to treat water? | - **Affordability.** - **Prioritization.** - **Perceived negative consequences** - **Time.** - **Knowledge** - **Attitudes/Beliefs** - **Accessibility** - **Resources/Hardware (**think about how this relates to the other probes) |
| How do you store your drinking water? | - **After treating?** - **Hardware.** - **Recontamination.** How do you get water from the storage container? - **Cleaning.** How often do people clean their drinking water storage containers? - **Time.** How long does the water stay in the storage container? Different times for different storage containers? |

| **Hygienic Play Area** | |
| --- | --- |
| Where do babies in the community play inside the family compound (outside the family dwelling)?  Inside the home/family dwelling?  Tell me about where babies play when they are outside the family compound? | - What age do children start playing outside the home? - Who helps take care of the baby during the day? Who watches the baby when they are playing? - Beliefs/Social norms Can you please describe a place for babies to play that is clean? Can you describe a place that babies may play that is unclean? - How do you define the difference between an unclean and clean place for babies to play? - What are things that could get babies sick? Why do children in the community get sick? - What are some ways CU2 in the community gets sick? - Why might it be good for babies to play in the yard (other unclean space)? Why might it be bad for children to play in the yard? Not have their own special place to play? - Does anyone ever talk about creating a safe, clean place for children to play? What do they say? - Are there materials in the community to create clean play areas for children? - Can they be accessed by community members? - How willing do you Do you think mothers would be willing to spend money to create a clean, safe play place for their child? - How willing do you think fathers would be willing to spend money to create a clean, safe play place for their children? - How would you create a clean area for your baby to play in? - How would mothers keep it clean? |

| **Deworming** | |
| --- | --- |
| What do you think about worms? | - When do you think worms are an issue/problem? (Specific ages, gender, etc) |
| What do you know that can be done to prevent worms? | - Who told you about this? |
| What do you know that can be done to treat worms? |  |
| Tell me if you think this medication has been given to mothers in the community? (Sample box for deworming medication) Do you know what it is used for? | - What does the de-worming medication do? Who should receive deworming medication? - Can women take deworming medication during pregnancy? Who does the mother learn about deworming from? Who does the mother trust? Is deworming medication okay for the baby? - Should pregnant women receive deworming medication? If yes, then when? - Should lactating women? If yes, then when? - Women of reproductive age? If yes, then when? - Children <1 year? If yes, then when? - Children >1year? If yes, then when - Who else should get treatment? - Where in the community can you get deworming medication? - What are barriers to participating in deworming? Availability? Cost? Accessibility? - Are there any specific days where you know deworming medication is being distributed? How do you know this? - Who usually brings the child to deworming days? |
| Would you be interested in attending support group sessions to talk about challenges women experience with other mothers and a counselor? | - Preferred frequency within a month for mothers to attend group sessions i.e. once a month, twice a month, every week - Preferred duration of the sessions for mothers i.e. half hour, one hour, hour and a half, two hours? - Likelihood to participate in a home visit session for your household i.e. very likely, attend some sessions, unlikely to attend? - Barriers to supporting participation - Recommendations |
| What would you like to learn about from a program focusing on pregnant women, mothers of young children, and young child health? What kind of role would you like to have in this program? | - Probe why and how. What is your interest in learning about 1) health and feeding practices, 2 how to maintain a clean environment for your baby; 3) water and sanitation practices that promote health - What would facilitate your participation in the program? - What are some barriers to participation? - What recommendations can you give to sustain positive practices in caring for children below 2 years? Pregnant mothers? |
| What would make this program successful? | - What would you like to see that will tell you the program is successful? |
| Before we end our time, I want to ask if you have any questions or other thoughts you would like to share with me. (Allow room for thoughts, questions, and comments)  Thank you so much for your time. | |

**Additional file 2**

**Mother/Caregiver of CU2 FGD Guide**

**Objectives:**

**Maternal Nutrition**

Objectives: Describe societal/cultural, environmental, family, and individual drivers/barriers of:

- Nutrition practices during pregnancy.
  - Domains: social/cultural norms; availability; accessibility/cost; priority; decision making; knowledge; agency; trusted sources of information

**Infant and Young Child Feeding**

Objectives: Describe societal/cultural, environmental, family, and individual drivers/barriers of:

- Hygienic, adequate, and appropriate young child complementary feeding behaviors.
  - Domains: social norms; social/cultural beliefs; availability; cost/accessibility; hardware availability; water availability; soap availability; drying rack availability; disagreement; knowledge; agency; ability

**Notes:**

- Exact wording of questions will change based on feedback from local research assistants.
- Snacks can be distributed either in the middle or end of the FGD. This is at the facilitator’s discretion.
- Prior to FGD, participants will be read the consent form and will be asked if they would like to voluntarily participate. Participants will be provided with numbers and must fill out demographic forms once they have consented. Full details to be found in the SOP

| Setting Ground Rules |
| --- |
| **Facilitator Notes:**  The facilitator should discuss ground rules with the participants. Facilitators should brief participants to:   - Put their phones on silent. If a participant needs to answer the phone, they should quietly leave the room. - Take care of their consents (place out of the way) - Say their number before sharing their thoughts and opinion - Speak one at a time - Be aware that their participation is voluntary. If they need/choose to leave at any point, that is allowed. - Be aware that there is no right or wrong answer. This is a participatory exercise and all are expected to participate. - Protect confidentiality. All responses shared in the FGD should not be shared with anyone outside of the group.   **Facilitator Notes:**  The facilitator can ask people to contribute their own ground rules to the group as well. Ask if people have any questions before beginning. |

| Opening Questions *Opening questions are intended to build rapport and gradually lead into primary/key questions* | | |
| --- | --- | --- |
| Opening Questions: Introductions | | |
| Each team should come up with a basic icebreaker or some closed questions that each person in the group can answer. ***Facilitator Script:*** Let’s go around and have each participant tell us   1. How many children they have? 2. What they might do on a typical day? (Make sure to cover this thoroughly) 3. Facilitators can use other icebreakers or conversation starters as appropriate | | |
| Food Card Sorting Activity **Materials:** Food cards. Include a few blank cards that could be filled out on the spot if participants feel like certain foods are missing.  **Facilitator Notes:** Show participants the food cards. Ask the participants if the foods on the cards are consumed in the community. Remove any cards that are not consumed. Ask the participants if any commonly consumed foods are missing from the cards. Use the blank cards to create cards for these foods (write the name of the food, these cards will not have pictures).  Ask participants to sort cards into the categories (see questions 1- Once the participants are finished sorting the cards ask them to discuss why they sorted them that way.  The note-taker should record the number of the food cards for each sort on the data collection sheet and add additional notes. | | |
| Sort by Source | | Produced by the home  - Gathered from the wild - Purchased in the market |
| Foods always available vs seasonal | | For seasonal, when are they available?  - What might affect availability? |
| Affordable/not affordable | | You may probe on “modern” vs “traditional” |
| Important for pregnant women to eat? Facilitator Notes: After they have created this pile, from the remaining cards, ask “Which are important for pregnant women not to eat?” | | Why? |
| Important for lactating women to eat? Facilitator Notes: After they have created this pile, from the remaining cards, ask “ Which are important that lactating women should not eat?” | | Why? |
| Reserved for men to eat | | Why? |
| Follow up Questions | | |
| Who decides what food should be purchased?Why?  - Control of financial resources with HH?   Who purchases food for the home? Why?  - If different than the person who controls finances, why?  Who decides what should be cooked?Why?What happens in a household if there is not enough food for everyone?Who gets food?  - Who does not get food? - Who gets fed first? Last? - What happens when there is no food and no resources to get more? | | |
| Lifeline Activity | | |
| ***Facilitator note****: Layout the Lifeline Activity Chart.* **Facilitator Script:** "Now I would like to know about when young children begin to eat these foods. As we go through the food cards, please lay the food cards down on the chart to reflect when they are given to children."   \|  \| \|  \| \|  \| \|  \| \|  \| \|  \| \|  \| \|  \| \|  \| \| --- \| --- \| --- \| --- \| --- \| --- \| --- \| --- \| --- \| --- \| --- \| --- \| --- \| --- \| --- \| --- \| --- \| \|  \| \|  \| \|  \| \|  \| \|  \| \|  \| \|  \| \|  \| \|  \| \| Birth \| \| 1 mo \| \| 3 mo \| \| 6 mo \| \| 9 mo \| \| 12 mo \| \| 18 mo \| \| 24 mo \| \|  \| \| | | |
| Which of these foods are typically given to infants shortly after birth or within the first week? | Why? | |
| When do families begin giving other foods? | Why? | |
| *NOTE – they may not put anything before 6 months and may say that they are not supposed to give anything before 6 months as that is the recommendation that is pushed and they may not want to identify as violating the recommendation; however if you note that nothing is being placed prior to 6months you can ask them whether they know of families that do give foods or drinks before 6 months They will likely say yes so you can ask when those families typically start giving other drinks/foods and which ones.* For each food placed before 6 months, **ask the reasons for why that food might be started at that time.** | | |
| If families were recommended by a health worker to give only breastmilk in the first 6 months, (that means not even water or very thin uji,) what challenges (related to exclusive breastfeeding) would they face? | How could those challenges be addressed? | |
| What would make it easier for families to give only breastmilk to infants for the first six months? |  | |
| Who in the household would be most important to support a mother to give breastmilk? | Why? | |
| Are there any foods that children can only eat for a certain amount of time? | Why? Which foods?  - When do they stop eating these foods? | |
| *Note for facilitator: For foods that are not started in the 6-9 month window but are delayed to later* ***ask about the different foods and why they are delayed to the indicated age*** *– this is especially critical for nutrient-dense foods such as eggs, meat, fruits, vegetables, legumes. If there are foods not included at all on the timeline ask why these foods are not included.* | | |
| If families were recommended by a health worker to give [XXX] beginning in the 6^th^/7^th^ month what challenges would they face? | *Go through each delayed food group – eggs, meat, peas/beans, nuts, fruits, vegetables – may be categories or maybe specific nutritious foods*}  - Social norms - Cost - Availability - Accessibility - Picky eater | |
| What would make it easier / more acceptable for families to give these foods to infants beginning in the 6^th^ or 7^th^ month? |  | |
| When does the child get their own food? |  | |
| When does the child eat from the family pot? |  | |
| Facilitator Script: Now I would like to talk a bit more about the foods given to infants 6-9 months of age – you have listed here uji (or other porridge, depending on what is listed). I would like to know more about how this food is prepared – | | |
| Can you describe for me how most families make this porridge? | Please talk through ingredients-how much liquid, how much flourWhat is typically added? | |
| How thick do families make their porridge for infants at this age? | Why?  - Instructions? Where did you learn? - From whom? - Availability? - Cost? | |
| (If thin/watery) if families were recommended by a health worker to give their infants thick porridge (use picture) beginning at the 6th/7th month what challenges would they face? | What would make it easier/more acceptable for families to give thicker porridge to infants beginning in the 6th or 7th month? | |
| About how many times in one day are infants in this community fed porridge? |  | |
| How would people feed the porridge to their infant? | Why? | |
| About how much are infants fed at each meal? | How do families know when their babies have had enough? | |
| Who is the main person to feed the child? | Who helps sometimes? | |
| Thank you. Now we’d like to talk a little about the preparation of food. You all said that _____________ is a typical food that is given to children under 2 (pick a cooked food). | | |
| When do mothers make this food? | Time of day  - Number of times a day (once and then stored? - Whether baby is hungry (how do they know) | |
| Can you walk me through all the steps that mothers take when they make this food? | Handwashing  - Washing of food (why? how?)_ - Boiling water/cooking food all the way - Cutting food - Dishes for preparation - Where the food is put when prepared? - Where the food is put if there is any leftover? - How to clean up after cooking? | |
| How would a person feed their baby/child this food? | Responsive feedingDifferent techniquesWith a spoonCover if baby takes a break from eating | |
| How would a mother clean up any dishes used in preparing the food? | Water (clean)?  - Soap? - Leaves? - Drying? - Location of washing?  Location of drying? | |
| Decision making and influencers | | |
| How do families decide which foods to give to infants and when to give them? | - Break this out by age – in the first week of life? - In the first 6 months? - After 6 months? | |
| Who in the family do mothers turn to for advice on how to feed young infants – for example, which foods to give, when to give those foods, and how much to feed? | - Why do they turn to those family members? | |
| Who do they go to in the community when they have questions about how to feed their young children? Why do they turn to those community members? | - Why do they turn to those community members? | |
| Where else might mothers learn about how to feed their infants? | - Friends/neighbors with young children, - media, - teachers, - religious leaders, - traditional birth attendants, - health care workers, etc | |
| Of all the ways mothers can learn about how to feed their infants, which ways do they prefer? | - Why? | |

| THRIVE program participation | |
| --- | --- |
| Would you be interested in attending support group sessions to talk about challenges women experience with other mothers and a counselor? | - Preferred frequency within a month for mothers to attend group sessions i.e. once a month, twice a month, every week - Preferred duration of the sessions for mothers i.e. half hour, one hour, hour and a half, two hours? - Likelihood to participate in a home visit session for your household i.e. very likely, attend some sessions, unlikely to attend? - Barriers to supporting participation - Recommendations |
| What would you like to learn about from a program focusing on pregnant women, mothers of young children, and young child health? What kind of role would you like to have in this program? | - Probe why and how. What is your interest in learning about 1) health and feeding practices, 2 how to maintain a clean environment for your baby; 3) water and sanitation practices that promote health - What would facilitate your participation in the program? - What are some barriers to participation? - What recommendations can you give to sustain positive practices in caring for children below 2 years? Pregnant mothers? |
| What would make this program successful? | - What would you like to see that will tell you the program is successful? |
| Before we end our time, I want to ask if you have any questions or other thoughts you would like to share with me. (Allow room for thoughts, questions, and comments)  Thank you so much for your time. | |

**Additional file 3**

FOCUS GROUP DISCUSSION GUIDE**: GRANDMOTHERS**

**Objectives:**

- Understand how grandmothers perceive infant and young child feeding (IYCF), water sanitation and hygiene (WASH), and deworming practices in targeted communities
- Understand the roles and responsibilities that grandmothers understand themselves to have related to IYCF, water sanitation, and hygiene, and deworming practices
- Determine current knowledge, attitudes, and practices on infant and young child feeding (IYCF), WASH, and deworming in the targeted communities

| Setting Ground Rules |
| --- |
| **Facilitator Notes:**  The facilitator should discuss ground rules with the participants. Facilitators should brief participants to:   - Put their phones on silent. If a participant needs to answer the phone, they should quietly leave the room. - Take care of their consents (place out of the way) - Say their number before sharing their thoughts and opinion - Speak one at a time - Be aware that their participation is voluntary. If they need/choose to leave at any point, that is allowed. - Be aware that there is no right or wrong answer. This is a participatory exercise and all are expected to participate. - Protect confidentiality. All responses shared in the FGD should not be shared with anyone outside of the group.   **Facilitator Notes:**  The facilitator can ask people to contribute their own ground rules to the group as well. Ask if people have any questions before beginning. |

| **Opening Questions** | |
| --- | --- |
| 1. Please tell us about a typical day in your life. From when you wake up to when you go to sleep? | - Responsibility: What are your activities and responsibilities? Why? How long have these been your responsibilities? - How do your responsibilities relate to those of other people in your household? - Value: How do your responsibilities contribute to the well-being of your household? Your community? Do members of the home value grandmother/father? How? How do grandmothers/fathers feel appreciated? - What do you do with any leisure time? - What do you enjoy most about your responsibilities? Least about your responsibilities? |
| 2. I’d like you to tell me about your responsibilities in taking care of a child from the time it is born to when it is 2 years. | - What are your responsibilities when the child is born? A few weeks of life? - When the child is growing up, from 6-12 months? - **Decision-making**. What kind of decisions do you make in your household? What kind of decisions may change when the mother is pregnant? When it is the first child? When the child grows? - **Time.** What times do fathers stay with the children? - **Socio-cultural Beliefs.** When you were growing up, what did you see as the role of fathers? What difference do you see now (from how things were when you were a child? - **Value.** How do people in the household value fathers? How do they feel when they share their experiences? How do people appreciate fathers? |
| Scenario: Now I want you to imagine a woman in your community named Emily. (For elder women, this may be someone like your daughter in law or daughter, for men, imagine Emily to have similar experiences to your wife or sister). | |
| Now I want you to imagine a woman named Emily. She is pregnant with a child. | - How does Emily’s diet change, if at all, while she is pregnant? - What foods might Emily eat that will be different than the rest of the family? Special foods? Taboo foods? - Does Emily eat more food than she would normally eat or less food than she would normally eat? Why? - Who prepares the food for her? - **Knowledge** How does Emily know which foods she fathers/grandmothers should/shouldn’t eat? - **Cost** Does cost define the foods that Emily will eat? - **Decision Making** Who decides who will eat which foods? Taboo foods? Special foods for certain people? How much food for certain people? - How will Emily’s diet change after she has the baby? While she is breastfeeding? - What would be the fathers/grandmothers role in assisting/supporting Emily during this time? |
| In the time right after Emily gives birth, how are she and the baby is taken care of? | - Where would Emily give birth? - Who would be with Emily to help her? - Who will tell her what to do and how to care for the baby? - How will she be cared for in the weeks after the baby is born? - When thinking about the responsibilities of the household as spoken about above, are there things that other people may do for Emily for some time? Who? For how long? - What would be the fathers’/grandmothers’ roles in assisting Emily during this time? |
| FOR GM ONLY: Once the baby is born, who helps Emily to feed the child? | - **Beliefs-Feeding?** - **Early Initiation/Skin to skin contact**: What happens between the time Emily’s baby is born and the time s/he is first put to the breast? How much time will pass? Cultural/religious/health care practices - Other than breastmilk, what else might Emily give her baby to eat/drink in the first few days of life? Why? - Other than breastmilk, what else might Emily give her baby in the first few months of life (honey, water, medicines, animal milk, thin porridge tea, etc.)? Why? What about if it is the dry season? When does this happen? - When will Emily begin giving her baby thin porridge? Why at that age? - Knowledge. How does Emily learn about how to feed her baby? Who advises her? Who teaches her? - **Perceived negative consequences-** What Do you all think are the consequences of giving only breastmilk to the child for the first six months of life – this means no water, animal milk, teas, or thin porridge at all. - **What challenges might Emily have giving only breast milk for the first six months?** How common it is for women in this community to have a hard time producing enough milk? How do women know they are not producing enough milk? How do women know their child is hungry? What strategies do women take to try to increase their breastmilk production? - **Time** Do women have time to breastfeed while maintaining their other responsibilities in the home? When the baby is still very young, a few months old, what are some reasons Emily might need help to feed her baby? (Work outside the home, school, Time due to household chores). - **Social Norms/Childcare** Who in the household is most likely to help Emily feed the baby when she needs help or is not available to feed the baby? What will they feed Emily’s baby (emphasize that the baby is only a few months old)? - What would be the fathers’/grandmothers’ role in assisting/supporting Emily during this time? |
| (GM Only) Now the baby is getting bigger… | - **Age** At what age will Emily’s baby begin to eat thick porridge or other semi-solid or soft foods? Why at this age? - **Social-cultural beliefs?**- other than breastmilk, what foods are needed to help a baby grow? When are babies first given these foods (at what age or developmental sign)? Why not earlier? What makes it possible for women to give these foods when they do? (Probe fruit, beans, eggs, and meat. When are they given to children? Why? ) - **Affordability**. How does cost play a role in determining what Emily’s baby will eat? - **Responsibility.** Who usually feeds Emily’s baby, now that s/he is a bit older (9-12 months). What happens if Emily is not available to feed the baby – she has gone to the field or traveled for the day. Who feeds Emily’s child? What will they feed the child? - **Perceived positive and negative consequences-**Is there a specific amount that will help the baby grow - What would be the fathers/grandmothers role in assisting/supporting Emily during this time? |
| **Thank you** very much for helping us think through the scenario of Emily and the child, now we’ll talk through some questions related to sanitation and hygiene in the community | |

| **Child Feces Disposal** | |
| --- | --- |
| **Facilitator Note:** At this point, you should refer back to the map of the compound that was made earlier to discuss the different places for child defecation and feces disposal. As you are going through these questions, ask people to show you on the compound map where children defecate. | |
| Where are all the places a CU2 might defecate? | **Facilitator instructions:** Ask   - Is there anywhere else you can think of? |
| What places are   - Most common? - Least common? - Best? - Safest? | - Why? |
| What influences where a child under 2 defecates? | - Age? - **Access** to latrine, - **Access to potty**, supporting tools - **Care of child**- Who is watching the child? When? Does this differ in the day? Night? - **Time of day?** - **Convenience** Locations that are easier to clean? Which are easier/harder to clean? - **Location**: Home/away? - **Perceived negative consequences:** Do you believe that child feces does not contain germs? Up until what age? - **Safety** |
| When are children old enough to use a toilet? | - Who teaches children? When? - Barriers? |
| If a family has access to a toilet, are there times when the child will not use the toilet? | - Why? |
| **Feces Disposal and Cleansing** | |
| What is done with child feces? Why are they handled this way?  **Facilitator Note:** As you go through the next set of probes asking about locations of child feces, ask the participants to show you these locations on the map of the compound from the earlier exercise.   - Cue to Action. Where is it normally put? - Where is it commonly disposed of? - Left in open. Why? How do you feel about this? - Why might people who have a toilet not dispose of children’s feces there? - How is it moved/transported (if children are not using the toilet)   - Clothes   - Hands   - Leaves   - Tools   - Potties - What kind of cleaning up happens after children defecate? | |
| What do you think is the safest way to dispose of child feces? |  |
| How do disposal practices of children’s feces change? | - Season - Time of day - Age of child (ability to walk) - Diarrhea |
| Who usually disposes of children’s feces in a household? | - Who else might help? - How might the person impact the disposal method? Child vs mother? |
| What are different ways to clean a child’s bottom after they defecate? | - Different materials? - Most common? - What do you think is safest? |
| Where might the water that is used to wash soiled clothes, rags, or potties be disposed of? |  |
| What about cloth or rags, what happens to these | - Washed/reused - Disposal |
|  |  |
| Who in the family decided whether to construct a toilet? Why? | - Barriers to toilet ownership - Available hand-ware? - Affordable? - Sustainability - Environment. Does the environment/land influence whether people may build a toilet or not? - Decision-making. Who decides whether to spend money to get a toilet? |
| Do some people in this community share toilets? Who? | - How many people? Who shares toilets? Who does not share toilets? |
| What are toilets usually made of? | - Why - Hardware? - Maintenance? - Time - Knowledge |
| Who is normally responsible for cleaning toilets? | - Why? - How often are the toilets cleaned? - Change when there are more people using? |

**Grandmother and child hand-washing**

| Can you describe how grandmothers typically wash their hands? |  |
| --- | --- |
| What about young children’s hands, are they washed in a similar manner as adults? Why or why not? | - Babies - Children under 2 - Who washes children’s hands? - When do children wash their own hands? |
| **Instructions for Facilitators:** As participants answer the next questions, in their notes, the notetaker makes a chart on a sheet of paper that lists the mother in one column, the baby in the second column, the times before activities in the third column, and the time after activities in the fourth column. The facilitator should prompt in the following order | |
| Can you tell me about all the events that occur in a day when it is necessary for grandmothers/fathers of CU2 to wash hands? | - **“**A grandmother will wash her hands *before* she/he _____________?” Why? - **“**A grandmother will wash her hands *after* she /he_____________?” Why |
| When are important times for grandmothers to use soap when handwashing? | - Are there certain events where a grandmother would use soap? Why? |
| When are times when it is necessary for CU2 to wash hands? | - **“**A grandmother will wash her childs’ hands *before* she _____________?” Why? - **“**A grandmother will wash her childs’ hands *after* she _____________?” Why - **Probe for**   - Eating (grandmother)   - Feeding a child   - Preparing food   - Farming   - After coming in contact with animals   - Defecating   - Washing a child’s bottom after defecation |
| When is it a good time for a grandmother to wash her/his____________ ‘s hands with soap   - Baby - CU2 | - Why? - Potential benefit? - Potential harm   - Self   - Others   - HH   - Community? |
| **Instructions for Facilitators** After the participants have listed important times for mothers and children under 2 to wash hands, The note-taker should put up the chart for mothers so they can see any differences. The facilitator should compare the two columns and probe on differences between the times mothers and babies wash hands and when they might use soap during a handwashing event. | |
| What are some times that you don’t wash your hands that you should? Could you tell us about the last time that happened? | - **Time.** Self, child. - **Affordability.** - **Accessible.** Where does the water in your community come from? - **Theft.** Soap - **Maintenance**. Water, station, etc. |
| Where do mothers wash their hands? | - **Different places** - **Hardware/facilities.** Basin, infrastructure, lake, tippy tap. - Near toilet? - Who else uses it? |
| Where does the water come from? | - **Primary Source?** - **Distance.** Where is the water source located? - **Alternative sources of water.** Seasonality - **Responsible?** - **How often replaced?** |
| Where does the soap come from? | - **Different kinds of soap** - **How often replaced** - **Affordable** - **Decision making.** Who decides whether to spend money on the soap? Why? |
| How common is it for toilets in this community to have water and soap near them? | - **Why/why not?** - **Barriers** |
| Thank you for discussing the practices related to sanitation and child feces disposal in your village. Now we will be moving on to discuss the ways in which mothers and other household members might be involved in programming that would teach them about some of the issues that we’ve discussed today. | |

| Would you be willing to support/allow the participation of mothers in your household to attend support group sessions to talk about challenges women experience with other mothers and a counselor? | - Preferred frequency within a month for mothers to attend group sessions i.e. once a month, twice a month, every week - Preferred duration of the sessions for mothers i.e. half hour, one hour, hour and a half, two hours? - Likelihood to participate in a home visit session for your household i.e. very likely, attend some sessions, unlikely to attend? - Barriers to supporting participation - Recommendations |
| --- | --- |
| What would you like to learn about from a program focusing on pregnant women, mothers of young children, and young child health? What kind of role would you like to have in this program? | - Probe why and how. What is your interest in learning about 1) health and feeding practices, 2 how to maintain a clean environment for your baby; 3) water and sanitation practices that promote health - What would facilitate your participation in the program? - What are some barriers to participation? - What recommendations can you give to sustain positive practices in caring for children below 2 years? Pregnant mothers? |
| What would make this program successful? | - What would you like to see that will tell you the program is successful? |

| Before we end our time, I want to ask if you have any questions or other thoughts you would like to share with me. (Allow room for thoughts, questions, comments)  Thank you so much for your time. |
| --- |

**Additional file 4**

**IN-DEPTH INTERVIEWS: Care Group Volunteers**

**Objectives:**

- Establish factors influencing uptake of practices around IYCF, WASH, and deworming
- Determine the capacity of Care Group Volunteers to implement IYCF, WASH, and deworming strategies in the target communities

| **PART 1 – HEALTH FACILITY INFORMATION** | |
| --- | --- |
| **A01**. **Researcher name:** | |
| **A02.** Date (dd/mm/yy): ____/_____/____ | **A03.** Start time ____: ___ am/pm  **A04.** End time ____: ___ am/pm |
| **A05.** **Care Group Volunteer name**: | |
| **A06** Number of years/ months served as Care Group Volunteer | **A07.** Care group volunteer sex:  1: Male 2: Female |
| **A6**. Number of neighbor women served_________ | **A7.**Number of villages served__________________ |

| 1. Please describe your role as a Care group volunteer? | - Activities, responsibilities - What are things you like most about your position? - What do you like least about your position? - How does your role relate to the THRIVE program? |
| --- | --- |
| 2. What are the most common health issues in the last one year that have occurred for   - pregnant mothers - Mother of CU2 - and children below 2 years   that have been attended to in the health facility? | - Pregnant women   - **Malnutrition** - Mothers of children below 2 years   - **Malnutrition** - Children below 2 years   - **Malnutrition**   - **Diarrheal disease** |
| 3. What information and services do you provide around   - maternal nutrition and healthy diet? - Infant and young child feeding? - WASH?   - Pur? - Deworming   - Pregnant mothers   - Mothers of CU2   - CU2 | - Information on promoting practices on IYCF, WASH maternal nutrition, and deworming - Specific messaging? Where does this come from? MOH? Other NGOs, APHIA Plus, other org? - When is it given? - How is it delivered? Which mode (IEC materials, training, ANC visits, dialogue days) - By whom? - To whom? - Follow-up? - Support? - Services within the health facility - In the community (outreach) - Supported by gov, NGOs, other organizations, specialists, - What kind of support do you receive? Financial support, technical support, capacity building, material support, supplies, transportation, personnel, etc. - Hospital/facility supports, programs for   - Early initiation   - Early skin to skin contact   - EBF - Sequence of care events immediately following delivery for the mother and infant (do these support early skin to skin and early initiation) - Is the facility baby-friendly? - What types of lactation support/breastfeeding support programs are offered (mother to mother support groups, classes, one on one lactation support)   - If any programs are these for all women or only HIV affected - Participation of fathers? - Participation of elder women? - What kind of engagement does the facility have with these people? - What have been their successes and challenges with engaging them? |
| 4. What are the key successes and challenges experienced by the health facility related to pregnant mothers and children below 2 years | - Successes   - IYCF   - WASH   - Deworming   - Maternal nutrition - Challenges   - IYCF   - WASH   - Deworming   - Maternal nutrition - Infrastructure & equipment - Personnel - Incentives - Health seeking individuals, - Promotion of health services - ECD spaces, - Available and functional management committee - Probe any specific indicators noted |
| 5. What is the relationship between community health volunteers and care group volunteers? | - Training-quality and quantity - Content of training- - Eligibility criteria? Preference towards men/women? Education level? - Motivation? What if anything do they receive for their time and efforts? - How many CGVs are supervised by CHV? What level of supervision occurs? - In what ways do you support the CHVs in their ability to deliver services? - How are health messages given by CGV tailored to match services/materials available in your facility? - What kind of relationships do HW have with CGVs? |
| 6. What are the roles of CGV in promoting the uptake of practices for appropriate   - Maternal nutrition - IYCF, - WASH, - deworming? | - What are their strengths in promoting the uptake of these practices? - What challenges do they experience? - What can be done to support CGVs in this role by the CHVs? By partners? |
| **Maternal Nutrition Practices** | |
| 7. Please tell us about what influences the uptake of key practices on maternal nutrition during pregnancy? | - **Social/cultural norms**-specific food to be eaten or avoided, specific amounts to be eaten - **Attitudes**- - **Knowledge**-what foods to eat and how much to eat during pregnancy - **Affordability**-cost too much - **Availability**-might not be available at certain times (though not too much variation) - **Accessibility**-may not be able to access it because it is too far, not allowed to go to market, etc. - **Priority**-other family members get more food, - **Decision-making** other family members decide what food to buy/eat - **Lack of agency**-cannot make choices about what she will/can eat - **Trusted sources of information**- Who do mothers speak to regarding their diet during pregnancy? About which foods and how much? Are there conflicting sources of information? Who does she trust most and why? - **Community support, institutional support**   - What changes in practice have been observed in the last two years for pregnant mothers? Mothers of children below 2 years? What has contributed to the change (s)?   What challenges are experienced in caring for pregnant mothers? Mothers of children below 2 years? |
| 8. What is your role in promoting the uptake of maternal nutrition practices? | - Role in promoting practices on maternal nutrition - What practices relating to maternal nutrition do you encourage people to do? - Why do you encourage them to do that? - What guides your knowledge/beliefs around these practices? - Relate any relationship noted in question #1 to this, if relevant. |
| 9. In your view what influences the uptake of practices about exclusive breastfeeding? Care for babies? | - **Beliefs- Colostrum** Is colostrum fed to babies? What do people think about colostrum? - **Beliefs-skin to skincare:** Is baby given to mother directly after birth? Why? Why not? - **Beliefs-washing breasts before baby nurses** - **Beliefs- first feeding** should the baby be given anything (honey, water, medicine, tea, etc) before nursing for the first time? - **Beliefs-Feeding in the first days of life?** What should children eat in the first few days of life? - **Beliefs-feeding in the first weeks of life?-** - **Lack of agency-**“care of”, feeding of a baby is taken over from the mother by other people (who?) - **Trusted sources of information-**Who informs mother on what to do? Who informs the mother related to colostrum? - **Insufficient milk**- How common is insufficient milk? - **Beliefs-insufficient milk:** Why do mothers think they have insufficient milk? What may be causing mothers to think that they have insufficient milk? - **Beliefs-exclusive breastfeeding:** Do they think the mothers would be able to only give breastmilk for the first 6 months? What would mothers need to be able to do this? - **Attractiveness-**breasts falling because of breastfeeding? - **Age-**Do certain aged people feel differently about breastfeeding? (teen mothers) - **Time/Availability-**Are mothers available to breastfeed their child? Why? Why not? - **Perceived positive consequences-**may think that giving other foods (water, tea, etc) may be beneficial to a child’s health - **Perceived negative consequences-**May not be viewed as serious not to feed child other foods |
| 10. In your view what influences the uptake of practices about IYCF? Hygiene-related IYCF? | - **Knowledge**-what to feed the child? How much? When? Diversity? - **Beliefs- water:** When should water be introduced? - **Beliefs-animal milks:** When should animal milk first be introduced? - **Beliefs-porridge:** When should porridge first be introduced? - **Trusted source of knowledge**- Who informs young mothers on what they should do? Whose advice are they most likely to follow? Disagreement between family members about what is “right”? - **Attitudes-** - **Social-cultural beliefs**-what foods are needed when and why? What makes it possible for women to give food when they do and why? - **Affordability**-cost too much - **Availability**-might not be available at certain times (though not too much variation) - **Accessibility**-may not be able to access it because it is too far, not allowed to go to market, etc. - **Lack of ability**-other person buys food or controls $, decides what food to buy - **Perceived positive and negative consequences-**supposed to give a certain amount, - **Hygiene**-drying rack? Water for cooking? Soap for washing dishes (available and affordable)? Does not know how to clean/store dishes or food in a hygienic manner? Does not know how to prepare or introduce foods in a hygienic manner? Handwashing station? (where and what is used for handwashing?) - **Community support, institutional support** - What changes in practice have been observed in the last two years for pregnant mothers? Mothers of children below 2 years? What has contributed to the change? - What challenges are experienced in caring for pregnant mothers? Mothers of children below 2 years? |
| 11. What is your role in promoting the uptake of exclusive breastfeeding and IYCF practices? | - Role in promoting practices   - Care of baby directly after birth?   - Exclusive breastfeeding?   - Complementary feeding - What practices relating to maternal nutrition do you encourage people to do? - Why do you encourage them to do that? - What guides your knowledge/beliefs around these practices? |
| 12. In your view what influences the uptake of practices about WASH? | - **Social norms related** to handwashing, water treatment, and latrine use? - **Knowledge/Attitudes-**related to the use of safe water, water treatment, handwashing at critical times, proper handwashing steps, water storage, child fecal disposal, - **Accessibility**-water, water treatment products, soap, latrine, Water storage products - **Affordability**-water treatment products, water, soap, hardware for hand-washing stations, hardware for latrines, and latrines, safe water storage containers - **Environment**-soil (does it support latrine structure), presence of animals within the compound? Which animals? (Hygienic play area) - **Cultural beliefs** about water treatment, and latrine use? - **Decision making**-who decides how to prioritize HH costs and expenditures related to WASH? - **Perceived negative consequences**-lack of understanding for potential neg consequences for poor child feces disposal, unhygienic play area, handwashing - **Prioritization**-how to decide what is the most important thing to address… - **Policy**-government guidelines on hand-washing and sanitation (CLTS)- - **Support incentives** through government, development organizations, etc |
| 13. What is your role in encouraging practices related to   - Water - Sanitation   Hygiene | - What practices relating to   - Water?   - Sanitation?   - Hygiene?   do you encourage people to do?   - Why do you encourage them to do that? - What guides your knowledge/beliefs around these practices? - Relate any relationship noted in question #1 to this, if relevant. |
| 14. In your view, what influences the uptake of practices about deworming? | - **Knowledge/Attitudes**-knowing when to take deworming pills, know you should take, and what children should take, present programs (pregnant women, CU5, primary school children) - **Acceptability**-is this accepted within the community? Do most people do this? - **Affordability**-for women post-pregnancy - **Access**-distance to the health facility, mobility of women - **Perceived negative consequences**-side effects are worse than potential positive impacts, - **Perceived positive consequences**-are people aware of benefits? - **Personal concern about** taking medication during pregnancy - **Preference/social-cultural norms**-prefer to use traditional medicine for deworming? - **Social-cultural norms**-Perception that deworming is something only critical for children, not adults |
| 15. What is your role in encouraging practices related to deworming of   - Pregnant women - Mothers of CU2 - CUs | - What practices relating to deworming do you encourage people to do? - Why do you encourage them to do that? - What guides your knowledge/beliefs around these practices? |
| **Closing Questions** | |
| **Recommendations** | |
| 16. Can you tell us what you know about THRIVE/Mercy Orphans/Homa Hills Community Development Organization? | - Programming - Messaging - How are they working with the health facilities |
| 17. Can you give me some examples of other health or community development programs that have happened here in the last 5 years? | - What has characterized successful behavior change programs that have happened previously? Why? - Which were unsuccessful? Why? - What did the successful programs do differently that made them a success |
| 18. What would ensure that positive outcomes from teaching/learning about caring for children under 2 years would continue? |  |
| 19. What would ensure that positive outcomes from teaching/learning about caring for children under 2 years would be incorporated into everyday practice? |  |
| Before we end our time, I want to ask if you have any questions or other thoughts you would like to share with me. (Allow room for thoughts, questions, comments)  Thank you so much for your time. | |

**Additional file 5**

FOCUS GROUP DISCUSSION GUIDE**: FATHERS**

**Objectives:**

- Understand how fathers perceive infant and young child feeding (IYCF), water sanitation and hygiene (WASH), and deworming practices in targeted communities
- Understand the roles and responsibilities that fathers understand themselves to have related to IYCF, water sanitation, and hygiene, and deworming practices
- Determine current knowledge, attitudes, and practices on infant and young child feeding (IYCF), WASH, and deworming in the targeted communities

| Setting Ground Rules |
| --- |
| **Facilitator Notes:**  The facilitator should discuss ground rules with the participants. Facilitators should brief participants to:   - Put their phones on silent. If a participant needs to answer the phone, they should quietly leave the room. - Take care of their consents (place out of the way) - Say their number before sharing their thoughts and opinion - Speak one at a time - Be aware that their participation is voluntary. If they need/choose to leave at any point, that is allowed. - Be aware that there is no right or wrong answer. This is a participatory exercise and all are expected to participate. - Protect confidentiality. All responses shared in the FGD should not be shared with anyone outside of the group.   **Facilitator Notes:**  The facilitator can ask people to contribute their own ground rules to the group as well. Ask if people have any questions before beginning. |

| **Opening Questions** | |
| --- | --- |
| 1. Please tell us about a typical day in your life. From when you wake up to when you go to sleep? | - Responsibility: What are your activities and responsibilities? Why? How long have these been your responsibilities? - How do your responsibilities relate to those of other people in your household? - Value: How do your responsibilities contribute to the well-being of your household? Your community? Do members of the home value father? How? How do fathers feel appreciated? - What do you do with any leisure time? - What do you enjoy most about your responsibilities? Least about your responsibilities? |
| 2. I’d like you to tell me about your responsibilities in taking care of a child from the time it is born to when it is 2 years. | - What are your responsibilities when the child is born? A few weeks of life? - When the child is growing up, from 6-12 months? - **Decision-making**. What kind of decisions do you make in your household? What kind of decisions may change when the mother is pregnant? When it is the first child? When the child grows? - **Time.** What times do fathers stay with the children? - **Socio-cultural Beliefs.** When you were growing up, what did you see as the role of fathers? What difference do you see now (from how things were when you were a child? - **Value.** How do people in the household value fathers? How do they feel when they share their experiences? How do people appreciate fathers? |
| Scenario: Now I want you to imagine a woman in your community named Emily. (For elder women, this may be someone like your daughter in law or daughter, for men, imagine Emily to have similar experiences to your wife or sister). | |
| Now I want you to imagine a woman named Emily. She is pregnant with a child. (For both) | - How does Emily’s diet change, if at all, while she is pregnant? - What foods might Emily eat that will be different than the rest of the family? Special foods? Taboo foods? - Does Emily eat more food than she would normally eat or less food than she would normally eat? Why? - Who prepares the food for her? - **Knowledge** How does Emily know which foods she fathers/grandmothers should/shouldn’t eat? - **Cost** Does cost define the foods that Emily will eat? - **Decision Making** Who decides who will eat which foods? Taboo foods? Special foods for certain people? How much food for certain people? - How will Emily’s diet change after she has the baby? While she is breastfeeding? - What would be the fathers role in assisting/supporting Emily during this time? |
| (Fathers) Now I would like to ask you a bit about meals that are eaten with the family. | - Does everyone in the family eat together? Do certain people eat at different times? Why? - Do certain people eat different food than other people? - Who is served/eats first? Last? - **Responsibility**. Who determines what food will be bought? Why? - **Cost**. Who decides how much money will be spent on food? |
| **Mapping Activity. Hygienic Spaces for Children**  ***Facilitator Script:*** Now we would like to conduct an activity to map out some of the places where Emily’s baby may be spending time when it is not being held, or playing and especially as they are growing up. We would like to have a better idea of the spaces in the compound where children are spending time.  Facilitator Notes “We will work together to draw a map of a typical compound in your village. I would like you to think about/visualize a typical compound in the village. (The notetaker or the facilitator will now prompt the participants to give their feedback to help “draw” the map. All participants should be able to see the map as it is being drawn. Ideally, this should be on a table that everyone can gather around. begin with, can you draw a map of a “typical” compound in your village. **Facilitator note:** As the participants are drawing the map, encourage them to mark the placement of 1) house(s); 2) latrine; 3) chickens; 4) other animals; 5) sleeping area 6) cooking area; 7) storage areas for food and water, 8) washing area (especially for dishes).  ***Facilitator Script:*** Looking at the places you have marked out,   - Where might babies or children under 2 play? - Where might they be put down if the caregiver is doing some other kind of work? - (What other work would the caregiver do and where?) - Why are some spaces ok/safe for babies? Why are other spaces not ok/safe?   **Facilitator note:** If the participants do not identify spaces that are “clean” vs those that are “not clean”, probe on this.   - What kind of space do you think might be best for babies to play in? What are the barriers to creating these kinds of spaces? What do you feel these spaces would look like? What materials might be used? How do you think fathers and mothers would feel about spending money on creating these kinds of spaces? | |
| **Thank you** very much for helping us think through the scenario of Emily and the child, now we’ll talk through some questions related to sanitation and hygiene in the community | |

**SANITATION AND HYGIENE**

**Activity: Discussion of Water Sources and Sanitation Facilities**

| **Facilitator Note:** If it makes sense to use the household mapping to discuss where people urinate or defecate (for example) all people are talking about latrine use, then please do. But don’t start with this for all people, as there may be other spaces within the village/community where people are urinating and defecating that do not apply to the immediate compound/household map. | |
| --- | --- |
| Where do _____________typically urinate?   - - Men   - Women | - Women   - Pregnant   - After giving birth   - Lactating   - Menstruating |
| Where do ____________typically defecate?   - Men (old, young, boy) - Women (young unmarried, married, old woman | - Seasonal - Time of day? - Women   - pregnant,   - after giving birth,   - lactating,   - menstruating |
| Why do some people prefer toilets to urinate and defecate? Not prefer? | - **Benefits?** - **Negative Consequences**-what might happen if someone is not urinating and defecating in the toilet? Consequences for   - Individual   - Household   - Community - **Time**. Different preferences for toilet or open defecation depending on the time - **Place.** Home/out and about - **Sharing.** Are there people who might not share toilets with each other? People in a family who would use a toilet and people who would not? |
| What would people say if they knew someone was defecating in the open? |  |
| Who in the family decided whether to construct a toilet? Why? | - Barriers to toilet ownership - Available hand-ware? - Affordable? - Sustainability - Environment. Does the environment/land influence whether people may build a toilet or not? - Decision-making. Who decides whether to spend money to get a toilet? |
| Do some people in this community share toilets? Who? | - How many people? Who shares toilets? Who does not share toilets? |
| What are toilets usually made of? | - Why - Hardware? - Maintenance? - Time - Knowledge |
| Who is normally responsible for cleaning toilets? | - Why? - How often are the toilets cleaned? - Change when there are more people using? |

| **Household Water Treatment** | | |
| --- | --- | --- |
| What is the primary source of people’s drinking water? | - **Accessibility/distance** Where in relation to people’s homes? - **Alternative sources of water.** Seasonality. Different sources for different purposes. - **Responsible.** Who gets water? - **How often?** | |
| What do people do to make their water safe for drinking? | - **Treatment.** Bleach, Pur, water guard - **Boil.** If boil, how do you boil the water to make it safe? How do you know when it is safe? - **Filter.** - **Rainwater** | |
| What makes it difficult for people to treat water? | - **Affordability.** - **Prioritization.** - **Perceived negative consequences** - **Time.** - **Knowledge** - **Attitudes/Beliefs** - **Accessibility** - **Resources/Hardware (**think about how this relates to the other probes) | |
| How do you store your drinking water? | - **After treating?** - **Hardware.** - **Recontamination.** How do you get water from the storage container? - **Cleaning.** How often do people clean their drinking water storage containers? - **Time.** How long does the water stay in the storage container? Different times for different storage containers? | |
| Earlier, you said that people got drinking water from _________________?Where does the water for handwashing come from? | | - **Primary Source?** - **Distance.** Where is the water source located? - **Alternative sources of water.** Seasonality - **Responsible?** - **How often replaced?** |
| Where does the soap for handwashing come from? | | - **Different kinds of soap** - **How often replaced** - **Affordable** - **Decision making.** Who decides whether to spend money on the soap? Why? |
| How common is it for toilets in this community to have water and soap near them? | | - **Why/why not?** - **Barriers** |
| **Deworming** | | |
| What do you think about worms? | | - When do you think worms are an issue/problem? (Specific ages, gender, etc) |
| What do you know that can be done to prevent worms? | | - Who told you about this? |
| What do you know that can be done to treat worms? | |  |
| Tell me if you think this medication has been given to mothers in the community? (Sample box for deworming medication) Do you know what it is used for? | | - What does the de-worming medication do? Who should receive deworming medication? - Can women take deworming medication during pregnancy? Who does the mother learn about deworming from? Who does the mother trust? Is deworming medication okay for the baby? - Should pregnant women receive deworming medication? If yes, then when? - Should lactating women? If yes, then when? - Women of reproductive age? If yes, then when? - Children <1 year? If yes, then when? - Children >1year? If yes, then when - Who else should get treatment? - Where in the community can you get deworming medication? - What are barriers to participating in deworming? Availability? Cost? Accessibility? - Are there any specific days where you know deworming medication is being distributed? How do you know this? - Who usually brings the child to deworming days? |
| Thank you for discussing the practices related to sanitation and water treatment in your village. Now we will be moving on to discuss the ways in which mothers and other household members might be involved in programming that would teach them about some of the issues that we’ve discussed today. | | |

| Would you be willing to support/allow the participation of mothers in your household to attend support group sessions to talk about challenges women experience with other mothers and a counselor? | - Preferred frequency within a month for mothers to attend group sessions i.e. once a month, twice a month, every week - Preferred duration of the sessions for mothers i.e. half hour, one hour, hour and a half, two hours? - Likelihood to participate in a home visit session for your household i.e. very likely, attend some sessions, unlikely to attend? - Barriers to supporting participation - Recommendations |
| --- | --- |
| What would you like to learn about from a program focusing on pregnant women, mothers of young children, and young child health? What kind of role would you like to have in this program? | - Probe why and how. What is your interest in learning about 1) health and feeding practices, 2 how to maintain a clean environment for your baby; 3) water and sanitation practices that promote health - What would facilitate your participation in the program? - What are some barriers to participation? - What recommendations can you give to sustain positive practices in caring for children below 2 years? Pregnant mothers? |
| What would make this program successful? | - What would you like to see that will tell you the program is successful? |

| Before we end our time, I want to ask if you have any questions or other thoughts you would like to share with me. (Allow room for thoughts, questions, comments)  Thank you so much for your time. |
| --- |

**Additional file 6**

**WASH and Feeding Observation tool**

The observations are intended to give insight into the IYCF and WASH behaviors that caregivers practice at home. The first observation will take place over ½ a day (4 hours), and the second observation will take place over ~6hrs. The first observation will matter but serves primarily for the caregiver to become comfortable with the observer. The primary observation will take place over the 2^nd^ day. The observer is to note their observations on this and other sheets of paper. For the feeding events, including breastfeeding, food preparation, and complementary feeding, you may need to fill out more than one sheet, as you should be filling out for each incident/event that occurs. While the intent is to have you watching the child interact with the primary caregiver (assumed to be the mother), should the primary caregiver split from the child for any reason (to leave the compound for any period of time) your focus should continue to be the index child and the person who is caring for the child. In the written notes, the observer should make note of any significant change in behavior or environment from Day 1 to Day 2.

| **Background** |
| --- |
| Date of observation dd/mm/yyyy  / / |
| Name of fieldworker: |
| First and last name of the child being observed (if available) |
| Child’s age in months: Child’s sex: M F |
| The child is crawling? (Y) (N) walking? (Y) (N) Can sit on the ground independently? (Y) (N) |
| **Breastfeeding (only for children breastfeeding)** |
| 1. Is there a child breastfeeding? Y N 2. What time was the event?________________________ 3. In what way is the caretaker interacting with the child while they are breastfeeding? 4. When do they pay attention? 5. When do they not pay attention? 6. For how long does the infant breastfeed? Does the infant come off of the breast on his/her own or is the infant taken off the breast by the mother? 7. If the infant comes off the breast on his/her own, does the mother attempt to re-latch? 8. Did the caregiver or mother appear to face any challenges with breastfeeding?   7a. For the child:  7b. For the mother: |
| **Food Preparation for the Child** |
| 1. What time was the event__________ 2. What food(s) was/were prepared for the child? 3. Does the caretaker prepare the food fresh (i.e. Was the food made from scratch or did they use food that had been left over from previous meals? (Yes) (No) 4. If not, how long has the food for the child been sitting out? _______minutes 5. If not, how was the food stored before serving to the child? i.e. high, covered, in a cooking pot, in a separate dish? 6. If applicable, was the food thoroughly reheated before feeding to the child? 7. What time was the event__________ 8. What food(s) was/were prepared for the child? 9. Does the caretaker prepare food fresh? (Yes) (No) 10. If not, how long has the food for the child been sitting out? _______minutes 11. If not, how was the food stored before serving to the child? i.e high, covered, in a cooking pot, in a separate dish? 12. Was the food thoroughly reheated before feeding to the child? |
| **Complementary Feeding** |
| What was the time of the event?   1. Age of person feeding the child (generally): 2. Sex of person feeding the child: M F 3. During mealtimes when serving the food did the caregiver:    1. Wash her own hands before serving the child? (Yes) (No)    2. Wash the child’s hands (Yes ) (No)    3. Serve the child first (Yes ) (No) 4. Child eats a) by himself/herself b) with family members 5. How is the child fed during mealtimes (tick that which describes the majority of the feeding experience):    1. The child feeds themself without assistance from the caregiver ( )    2. The child mostly feeds themself but received help from a caregiver ( )    3. The child is fed mostly by the caregiver but sometimes feeds self ( )    4. The child is fed only by the caregiver (does not touch food or utensils) ( ) 6. Is the child served food on his/her own plate (Yes ) (No) 7. Which is used to feed the child / does the child use to eat (tick all that apply)?    1. Spoon or fork ( )    2. Caregivers or other person’s hands ( )    3. Child’s own hands    4. Bottle ( )    5. Other (specify) ( ) 8. Which best describes the caregiver during this feeding (tick that which applies the most)? 9. Caregiver is near (within 1 meter) the child and attentive to the child ( ) 10. Caregiver is near but not attentive to the child ( ) 11. Caregiver is not near the child but is still engaging the child verbally ( ) 12. Caregiver is not near the child and is doing something else / not engaged with child ( )   Food, dishes, and drinks served to a child:   1. Are certain food, dishes or drinks served only to the child (not to other members of the family)? 2. If so, which types of food, dishes, or drinks? 3. Are there certain foods that are not fed to the child, but are fed to other members of the family? (Look especially for protein-rich foods) 4. Is the child only served portions of the foods, or drinks that are served to the rest of the family, or are special foods prepared for the child? 5. What foods or drinks are served only to the child? 6. At any point during the entire household observation period does the child use a bottle with a nipple? (Yes ) (No) |
|  |
| How does Caregiver Motivate the Child to Eat?   1. How does the caregiver motivate the child to eat (if the caregiver does not talk to the child then indicate caregiver does not verbally motivate the child) What does the caregiver say to the child? What is the caregiver’s tone (i.e. encouraging, harsh, reprimanding)? 2. How does the caregiver physically motivate the child to eat (it does not physically motivate then indicate)? For example, does the caregiver use hand gestures/signals, play games, or by demonstrating how to eat? 3. During the meal, does the child ever refuse the food? YES NO 4. If yes, how does the caregiver respond? 5. During the meal, does the child have any other difficulties? YES NO 6. If yes, describe? 7. If yes, how does the caregiver respond? 8. Does the caregiver ever force-feed the child (i.e. holds the child’s mouth open and feeds the child)? YES NO 9. Does the child eat all of the food he/she is served? YES NO 10. Does the caregiver serve additional portions to the child?   What does the caregiver do with any leftovers?   1. How does the caregiver spend her time when the child is eating? 2. Other aspects related to the feeding 3. General observations about hygiene during food preparation and handling   After eating   1. Please describe what the caregiver does with any dishes used for eating, cooking, etc. when complete?   Are they washed? Where? How are they dried? Where are they stored?   1. Is food put away? How much time passed before the food was put away? 2. Is food covered? How much time passed before the food was covered? Please remark on any/all times this occurs throughout the observation? |
| **Water handling** |
| 1. What do people in the household use water for throughout the day? 2. Drinking (how much? Source?) 3. Cooking 4. Cleaning 5. Personal hygiene 6. Washing clothes 7. Watering plants 8. Watering animals 9. Other 10. Did you observe anyone fetching water?   Please describe. Who is responsible for doing this? What do they use to collect water?   1. Did you observe anyone treating the water used for cooking/drinking in any way?   (Boiling, water guard, sand filter, adding bleach, etc.) Who is practicing this behavior?   1. Did you observe anyone fetching water from a storage container? How did they do it? Did they use a utensil or dip their hands? 2. Did the child drink water? YES NO 3. Was the water treated in any way (sieved/filtered, boiled, chlorinated) before being given to the child? YES NO 4. IF yes, how was the water treated? 5. Was that water provided from a clean glass? YES NO |
| **Hygiene** |
| **Potential Caregiver HW event^1^ Handwashing** (Y/N)  **Soap**(Y/N)  **Dry**(air dry/clean cloth/dress/other)  1.  2.  3.  4.  6.  7.  8.  9.  *Note all handwashing events and what took place either before or after. If nothing specific, please note  **Potential Child HW event^1^ Handwashing** (Y/N)  **Soap**(Y/N)  **Dry**(air dry/clean cloth/dress/other)  1.  2.  3.  4.  6.  7.  8.  9.  *Note all handwashing events and what took place either before or after. If nothing specific, please note  **Other HW event^1^ Who? Handwashing** (Y/N)  **Soap**(Y/N)  **Dry**(air dry/clean cloth/dress/other)  1.  2.  3.  4.  6.  7.  8.  9.  *Note all handwashing events and what took place either before or after. If nothing specific, please note  **^1^ HW events example:** after toileting, before eating, after eating, after cleaning, before food preparation, before breastfeeding, after changing baby, after handling animals, before feeding child nothing specific |
| **General hygiene** |
| 1. Did you observe anyone putting water or soap out for handwashing? 2. Who in the household is wearing shoes? At what times are they wearing or not wearing shoes? 3. Is the child wearing shoes? |
| **Sanitation** |
| 1. Did you observe the child defecating? Is the child wearing a nappy or diaper? What does the caretaker do with the child’s feces after defecation? Look and comment on the location of disposal, method of disposal, and any cleaning up done for child, materials, or hands post clean-up. 2. Which members of the household did you observe using the toilet? How many people are using the toilet throughout the day? Who is using it? Is there any place to wash hands close to the toilet? Is there water and soap there? Did they do anything specific before or after using the toilet (put on shoes, clean, etc) 3. Did you observe anyone cleaning the toilet? Who? With what? |
| **Child play areas** |
| Where was the child in the compound?  Where are they playing/sitting/etc?  Is the area free from fecal contamination?  Is anyone playing with the child?  Is anyone watching the child throughout the day?  Was the child placed on a mat?  Was the child ever on an unimproved/dirt area?  Did the child come into contact with any animals?  What objects did you observe the child putting in her/his mouth?  Are there animals kept on the compound? Which ones? Where? Does the child come into contact with animals throughout the day? What type of contact? |
| **Child Care** |
| Who was taking care of the child throughout the day (by % time. Should add to 100%)  ________% Mother  ________%Father  ________%Sibling (age)  ________%Grandparent  ________%Other____________ |

**Additional file 7**

**Household Structured survey**

**ALL QUESTIONS ARE TO BE ADDRESSED TO MOTHERS WITH A CHILD < 24 MONTHS OF AGE**

**SEE THE ELIGIBILITY QUESTION BELOW TO VERIFY THE AGE OF BIRTH FOR THE WOMAN’S YOUNGEST CHILD**

| **IDENTIFICATION** | | | | | | | | | | | | | | |  |  |
| --- | --- | --- | --- | --- | --- | --- | --- | --- | --- | --- | --- | --- | --- | --- | --- | --- |
| Community Name: | | | | |  | | | | | | | | | |  |  |
| Name of Interviewer: | | | | |  | | | | | | | | | |  |  |
|  | | 1 | | | 2 | | | Final Visit | | | | | | |  |  |
| Interview date | | ___/___/___  day/mnt/year | | | ___/___/___  day/mnt/year | | | *For Supervisor* | | | | | | |  |  |
|  |  |  |  |  |  |  |  | Day | | | | |  |  |  |  |
|  |  |  |  |  |  |  |  | Month | | | | |  |  |  |  |
|  |  |  |  |  |  |  |  | Year | | |  |  |  |  |  |  |
| Result Code* | |  | | |  | | | Result Code | | | | |  | |  |  |
| *Result Codes: | | | | | | | | | | | | | | |  |  |
| 1. Completed | 2. Postponed | | | 3. Respondent not at Home | | | 4. Refused | | | 5. Other (Specify):___________________ | | | | |  |  |
| Form checked by | | | Supervisor Name: | | | | | | Supervisor Signature: | | | | | |  |  |
| Name of Data Entry | | | 1.  2. | | | | | | Date: ___/___/____  Date: ___/___/____ | | | | | |  |  |
| **ELIGIBILITY QUESTION – VERIFICATION OF AGE OF YOUNGEST CHILD** | | | | | | | | | | | | | | |  |  |
| E1 **[READ:]** What is the date of birth of your youngest child or the youngest child you care for?  **[ASK TO SEE ANC BOOKLET, CHILD WELFARE CARD, OR BIRTH CERTIFICATE, IF AVAILABLE.**  **INTERVIEW THE WOMAN ONLY IF CHILD WAS BORN BETWEEN July 2014 AND June 2016, OTHERWISE, END THE INTERVIEW]** | | | | | | Date of birth of youngest child……… ____/____/______  DD / MM / YYYY | | | | | | | | |  |  |
| E2 What is your relationship with the child? | | | | | | Biological mother………………………………… | | | | | | | | | 1 | →consent |
|  |  |  |  |  |  | Grandmother…………………………………….. | | | | | | | | | 2 |  |
|  |  |  |  |  |  | Aunt……………………………………………….. | | | | | | | | | 3 |  |
|  |  |  |  |  |  | Older Sibling……………………………………… | | | | | | | | | 4 |  |
|  |  |  |  |  |  | Other relative…………………………………….. | | | | | | | | | 8 |  |
| E3 Is the biological mother of this child alive? | | | | | | Yes………………………………………………… | | | | | | | | | 1 |  |
|  |  |  |  |  |  | No…………………………………………………. | | | | | | | | | 2 | →E6 |
|  |  |  |  |  |  | Do not know/Refused to Answer | | | | | | | | | 99 | →E6 |
| E4 Does the child’s mother live with him or her in this household? | | | | | | Yes………………………………………………… | | | | | | | | | 1 |  |
|  |  |  |  |  |  | No…………………………………………………. | | | | | | | | | 2 | →E6 |
| **E5 - [ASK TO INTERVIEW THE BIOLOGICAL MOTHER OF THE CHILD IF SHE IS ALIVE]** | | | | | | | | | | | | | | |  |  |
| E6 Do you regularly take care of and feed the child?  *?* | | | | | | Yes………………………………………………… | | | | | | | | | 1 | →Consent |
|  |  |  |  |  |  | No…………………………………………………. | | | | | | | | | 2 |  |
| **[IF THE BIOLOGICAL MOTHER IS NOT ALIVE OR DOES NOT LIVE IN THE HOUSEHOLD, ASK TO INTERVIEW THE PERSON WHO REGULARLY TAKES CARE OF AND FEEDS THE CHILD.]** | | | | | | | | | | | | | | |  |  |

Conduct Informed Consent

| **NO.** | **QUESTIONS AND FILTERS** | | **CODING CATEGORIES** |  | **SKIP TO** | |  |
| --- | --- | --- | --- | --- | --- | --- | --- |
| **SECTION 1 - RESPONDENT BACKGROUND CHARACTERISTICS** | | | | | | | |
| 101 | What is your highest level of formal education? | | No formal schooling ………………………………… | 1 |  | |  |
|  |  |  | Some primary school……………….……………….. | 2 |  | |  |
|  |  |  | Completed primary school………….………………. | 3 |  | |  |
|  |  |  | Completed secondary…………..…………………… | 4 |  | |  |
|  |  |  | Beyond secondary…………...……….……………... | 5 |  | |  |
| 102 | In what language do you feel most comfortable communicating? | | Luo.……………………………………….. | 1 |  | |  |
|  |  |  | Kiswahili………………………………………… | 2 |  | |  |
|  |  |  | English……………………………………….. | 3 |  | |  |
|  |  |  | OTHER………………………………………………… | 88 |  | |  |
| 103 | What religion do you practice? | | Christianity | 1 |  | |  |
|  |  |  | Islam…………………………………………………. | 2 |  | |  |
|  |  |  | African Traditional Religion………………………… | 3 |  | |  |
|  |  |  | None…………………………………………………… | 4 |  | |  |
|  |  |  | Other………………………………………………….. | 8 |  | |  |
| 104 | Who is the head of this household | | Mother (Respondent)…………………………………. | 1 |  | |  |
|  |  |  | Husband/ Partner……………………………………… | 2 |  | |  |
|  |  |  | Respondent’s Mother…………………………………. | 3 |  | |  |
|  |  |  | Respondent’s Mother-in-law…………………………. | 4 |  | |  |
|  |  |  | Respondent’s Father…………………………………. | 5 |  | |  |
|  |  |  | Respondent’s Father-in-law………………………….. | 6 |  | |  |
|  |  |  | Other……………………………………………………. | 88 |  | |  |
| 105 | Is the biological father of your youngest child alive? | | Yes…………………………………………………….. | 1 |  | |  |
|  |  |  | No……………………………………………………… | 2 |  | |  |
|  |  |  | Do not know/No response…………………………… | 99 |  | |  |
| 106 | Does the biological father live in the household? | | Yes…………………………………………………….. | 1 |  | |  |
|  |  |  | No……………………………………………………… | 2 |  | |  |
|  |  |  | Do not know/No response…………………………… | 99 |  | |  |
| 107 | Do you work outside of the home to earn money? | | Yes…………………………………………………….. | 1 |  | |  |
|  |  |  | No……………………………………………………… | 2 |  | |  |
|  |  |  | Do not know/No response…………………………… | 99 |  | |  |
| 108 | Who primarily takes care of [NAME] when you are away from home?  **[DO NOT READ RESPONSES. SELECT ONLY 1 RESPONSE** | | Mother (Respondent)…………………………………. | 1 |  | |  |
|  |  |  | Husband / Partner…………………………………….. | 2 |  | |  |
|  |  |  | Neighbors/Friends……………………………………. | 3 |  | |  |
|  |  |  | The older sibling of the child……..…………………………… | 4 |  | |  |
|  |  |  | Other relative of child…………………………………. | 5 |  | |  |
|  |  |  | Other…………………………………………………… | 88 |  | |  |
| **SECTION 11 –SURVEY QUESTIONS** | | | | | | | |
| 1001 | What is the main/primary source of water for drinking and cooking in the household?  **[SELECT ONLY 1 RESPONSE]** | Piped water into dwelling…………………………….. | | 1 | | →1104 | |
|  |  | Piped water to yard/plot/compound……………… | | 2 | | →1104 | |
|  |  | Public tap/standpipe………………………………… | | 3 | |  | |
|  |  | Tubewell/borehole…………………………………… | | 4 | |  | |
|  |  | Protected dug well…………………………………….. | | 5 | |  | |
|  |  | Unprotected dug well………………………………….. | | 6 | |  | |
|  |  | Protected spring………………………………………… | | 7 | |  | |
|  |  | Unprotected spring……………………………………. | | 8 | |  | |
|  |  | Rainwater collection…………………………………… | | 9 | |  | |
|  |  | Bottled water…………………………………………… | | 10 | |  | |
|  |  | Cart or tanker truck with small tank/drum………… | | 11 | |  | |
|  |  | Surface water (river, dam, lake, pond, stream)…….. | | 12 | |  | |
|  |  | Do not know / No response………………………….. | | 99 | |  | |
| 1002 | Where is this main source of water located? | In the dwelling/house…………………………………. | | 1 | | →1104 | |
|  |  | In the yard or compound……………………………… | | 2 | | →1104 | |
|  |  | Outside of the yard or compound…………………… | | 3 | |  | |
|  |  | Do not know/No response | | 99 | |  | |
| 1003 | How many minutes does it take to go there, get water, and come back? | Minutes…………………………  Do not know / No response………………………….. | | 999 | |  | |
| 1004 | What do you use this source for? | Drinking………………………………………………1  Cooking………………………………………….......2  Personal Hygiene………………………………… ..3  Cleaning and washing………………………………4  Agriculture……………………………………………5  Animals……………………………………………….6 | |  | |  | |
| 1005 | How many months is this source *not* available | ________________________months | |  | |  | |
| 1006 | What is your alternative source of water  **[SELECT ONLY 1 RESPONSE]** | Piped water into dwelling…………………………….. | | 1 | | →1104 | |
|  |  | Piped water to yard/plot/compound……………… | | 2 | | →1104 | |
|  |  | Public tap/standpipe………………………………… | | 3 | |  | |
|  |  | Tubewell/borehole…………………………………… | | 4 | |  | |
|  |  | Protected dug well…………………………………….. | | 5 | |  | |
|  |  | Unprotected dug well………………………………….. | | 6 | |  | |
|  |  | Protected spring………………………………………… | | 7 | |  | |
|  |  | Unprotected spring……………………………………. | | 8 | |  | |
|  |  | Rainwater collection…………………………………… | | 9 | |  | |
|  |  | Bottled water…………………………………………… | | 10 | |  | |
|  |  | Cart or tanker truck with small tank/drum………… | | 11 | |  | |
|  |  | Surface water (river, dam, lake, pond, stream)…….. | | 12 | |  | |
|  |  | Do not know / No response………………………….. | | 99 | |  | |
| 1007 | How many months is this source *not* available | months | | _____ | |  | |
| 1008 | What do you use this source for? | Drinking………………………………………………1  Cooking………………………………………….......2  Personal Hygiene………………………………… ..3  Cleaning and washing………………………………4  Agriculture……………………………………………5  Animals……………………………………………….6 | |  | |  | |
| 1009 | Do you have secondary sources of water? | Yes………………………………………………………1  No……………………………………………………….2 | |  | |  | |
| 1010 | What are these other sources of water?  **[SELECT MULTIPLE]** | Piped water into dwelling…………………………….. | | 1 | | →1104 | |
|  |  | Piped water to yard/plot/compound……………… | | 2 | | →1104 | |
|  |  | Public tap/standpipe………………………………… | | 3 | |  | |
|  |  | Tubewell/borehole…………………………………… | | 4 | |  | |
|  |  | Protected dug well…………………………………….. | | 5 | |  | |
|  |  | Unprotected dug well………………………………….. | | 6 | |  | |
|  |  | Protected spring………………………………………… | | 7 | |  | |
|  |  | Unprotected spring……………………………………. | | 8 | |  | |
|  |  | Rainwater collection…………………………………… | | 9 | |  | |
|  |  | Bottled water…………………………………………… | | 10 | |  | |
|  |  | Cart or tanker truck with small tank/drum………… | | 11 | |  | |
|  |  | Surface water (river, dam, lake, pond, stream)…….. | | 12 | |  | |
|  |  | Do not know / No response………………………….. | | 99 | |  | |
| 1011 | Is the water stored in the interior of the home? | Yes…………………………………………………….. | | 1 | |  | |
|  |  | No……………………………………………………… | | 2 | |  | |
|  |  | There is no drinking water stored……………………. | | 3 | |  | |
|  |  | Do not know / No response………………………….. | | 99 | |  | |
| 1012 | Does the container have a lid? | Yes………………………………………………………1  No……………………………………………………….2 | |  | |  | |
| 1013 | How does the household treat water to make it safe to drink? | Let it stand and settle / Sedimentation……………… | | 1 | |  | |
|  |  | Strained it through cloth…………………… | | 2 | |  | |
|  |  | Boiled………………………………………………… | | 3 | |  | |
|  |  | Added bleach/chlorine……………………………… | | 4 | |  | |
|  |  | Water filter (Ceramic, sand, composite)…………… | | 5 | |  | |
|  |  | Solar disinfection (see a bottle on the roof)…………………… | | 6 | |  | |
|  |  | Other……………………………………………………. | | 88 | | →1015 | |
|  |  | Do not know/Not clear)…..………………………. | | 99 | | →1015 | |
| 1014 | Was the water used today for drinking treated? | Yes………………………………………………………1  No……………………………………………………….2 | |  | |  | |
| 1015 | What happens with the stools of babies and young children in the household who do not use the toilet facility?  **[DO NOT READ RESPONSES. RECORD ALL THAT YOU SEE]** | Thrown in toilet or latrine……………………………… | | 1 | |  | |
|  |  | Buried in the yard……………………………………… | | 2 | |  | |
|  |  | Not disposed of/Left on the ground…………………. | | 3 | |  | |
|  |  | Do not know/No response……………………………. | | 99 | |  | |

| **OBSERVATIONS** | | | | |
| --- | --- | --- | --- | --- |
| 1101 | What animals are observed in the compound? | Poultry…………………………………………….. | 1 |  |
|  |  | Dogs……………………………………………………..Cats……………………………………………………….Goats……………………………………………………  Cattle……………………………………………………... | 2  3  4  5 | →1104 |
| 1102 | Are the poultry kept contained in a pen on the compound? | Yes…………………………………………………….. | 1 |  |
|  |  | No……………………………………………………… | 2 |  |
| 1103 | Are the animals able to go in and out of the house? | Yes…………………………………………………….. | 1 |  |
|  |  | No……………………………………………………… | 2 |  |
| 1104 | Is there a trash pit in the yard/compound? | Yes…………………………………………………….. | 1 |  |
|  |  | No……………………………………………………… | 2 |  |
| 1105 | Is there food left uncovered? | Yes……………………………………………………1  No……………………………………………………….2 |  |  |
| 1106 | What type of toilet is present? | No facilities or bush or field……………………………. | 1 | →1111 |
|  |  | Flush to Septic tank……………………………………. | 2 |  |
|  |  | Flush to pit latrine……………………………………… | 3 |  |
|  |  | Flush to somewhere else…………………………….. | 4 |  |
|  |  | Ventilated improved pit latrine (VIP) ……………….. | 5 |  |
|  |  | Pit latrine with slab……………………………………. | 6 |  |
|  |  | Pit latrine without slab / open pit………………………. | 7 |  |
|  |  | Composting toilet……………………………………… | 8 |  |
|  |  | Bucket toilet……………………………………………. | 9 |  |
|  |  | Hanging toilet / Hanging latrine………………………. | 10 |  |
|  |  | Other……………………………………………………. | 88 |  |
| 1107 | Is this toilet shared with other households? | Yes…………………………………………………….. | 1 |  |
|  |  | No……………………………………………………… | 2 |  |
| 1108 | Does the toilet have a roof? | Yes…………………………………………………….. | 1 |  |
|  |  | No……………………………………………………… | 2 |  |
| 1109 | Are there obvious signs of use of the toilet facility  ? (footpath, hole, smells) | Yes…………………………………………………….. | 1 |  |
|  |  | No……………………………………………………… | 2 |  |
| 1110 | Does the toilet have solid walls and a door that closes completely? | Yes…………………………………………………….. | 1 |  |
|  |  | No……………………………………………………… | 2 |  |
| 1111 | Is there a place to dry plates and cooking utensils? | Yes …………………………………………. | 1 |  |
|  |  | No……………………………………………. | 2 |  |
| 1112 | Is there an area where children can play that is separate from animals and other sources of fecal contamination? | Yes …………………………………………. | 1 |  |
|  |  | No……………………………………………………… | 2 |  |
| 1113 | Are there any visible signs of animal feces present in the compound or home? (walk around the compound once?) | Yes …………………………………………. | 1 |  |
|  |  | No……………………………………………. | 2 |  |
|  |  | Do not know/No response…………………………….. | 99 |  |
| 1114 | Are there any visible signs of human-animal feces present in the compound or home? (walk around the compound once?) | Yes, animal…………………………………………. | 1 |  |
|  |  | No……………………………………………. | 2 |  |
|  |  | Do not know/No response…………………………….. | 99 |  |
|  | **OTHER COMMENTS** |  |  |  |
