# Supplementary material for: Determinants of food preparation and hygiene practices among caregivers of children under two in Western Kenya: a formative research study
Source: BMC Public Health. 2022 Oct 6;22:1865. doi: 10.1186/s12889-022-14259-6 (PMC9535979; doi:10.1186/s12889-022-14259-6)
Supplement: Supplementary file 2 — Additional file 2. [file 12889_2022_14259_MOESM2_ESM.docx]

***Supplementary table.*** *Results summary of facilitators (+) and barriers (-) to food preparation and hygiene practices categorized by COM-B domains*

| **COM-B domain determinant definition** | **Handwashing with soap at key events** | **Washing food** | **Cooking and reheating food** | **Cleaning utensils and food preparation locations** | **Covering and storing food** |
| --- | --- | --- | --- | --- | --- |
| **Physical capability (Having physical skills, stamina and strength** | (+) Caregivers’ physical ability to wash and air-dry hands  (+) Caregivers’ physical ability to wash their children’s hands  (+) Caregivers’ physical ability to fetch water | (+) Caregivers’ physical ability to wash food | (+) Caregivers’ physical ability to cook food | (+) Caregivers’ physical ability to wash utensils | (+) Caregivers’ physical ability to cover and store food |
| **Psychological capability**  **(Capacity to engage in necessary thought process- comprehension and reasoning -presence/ lack of knowledge, skills, and**  **behavioral regulation)** | (+) Some caregivers demonstrated knowledge and skills of their hands and children’s hands  (+) Some caregivers demonstrated knowledge of critical times for handwashing  (-) Some caregivers demonstrated a lack of knowledge of some critical times for handwashing | (+) Some caregivers demonstrated knowledge and skills of washing kale before slicing  (-) Some caregivers demonstrated a lack of knowledge that fruits that required peeling and potatoes or cassavas needed to be washed before eating | (+) Some caregivers had knowledge and skills of cooking food thoroughly  (+) Some caregivers demonstrated knowledge of cooking some foods like kale in small portions to avoid spoilage  (-) Some caregivers believed in cooking food in large quantities  (-) Older siblings lacked knowledge on importance of reheating food before eating and feeding young child | (+) Some caregivers demonstrated knowledge and skills of washing and rinsing utensils 3 times  (+) Some caregivers demonstrated knowledge on benefits of sun drying utensils  (-) Some caregivers demonstrated a lack knowledge on possible microbial recontamination by wiping wet utensils with towel | (+) Some caregivers had knowledge and skills of covering food after cooking  (-) Some caregivers believed in storing food for >4 hours, even overnight |
| **Physical opportunity**  **(Environmental context and**  **resources, including time,**  **affordability of resources,**  **access, and enabling**  **environment)** | (+) Some caregivers used ash/salt when soap was not available  (+) Soap was available for purchasing nearby  (-) Some caregivers lacked soap because of affordability  (-) Some caregivers lacked water  (-) Distance to water source was far away  (-) Caregivers lacked handwashing station in food preparation area | (+) Some caregivers prioritized available water in the house for washing vegetables  (+) Basin for washing vegetables was available  (-) Some caregivers lacked water  (-) Distance to water source was far away | (+) Some caregivers had food available  (+/-) Firewood was available  (+/-) Water was available  (-) Time constraint due to work demands | (+) Caregivers had drying racks, crates, fences, and basins to dry utensils  (+/-) Soap was available for cleaning utensils  (+/-) Water was available  (-) Racks destroyed by animals  (-) Height of racks did not deter animal access | (+) Caregivers had different items for covering food like plates, cooking pot, and metallic covers  (+) Caregivers used varied ways of storing food: hanging on the rope, keeping in cupboards, storing in thermos |
| **Social opportunity (Include**  **social and**  **cultural norms, and interpersonal**  **influence)** | (-) Women lacked monetary decision-making power; unable to prioritize soap for handwashing | (-) Social norm of not washing certain vegetables  (-) Social norm of men purchasing food so caregivers lacked control of separating food (uncooked fish, meat from fruits and vegetables) | (+/-) Women and older children shared roles of cooking  (-) Social cultural norms of certain foods (githeri) not being reheated  (-) Social norm of “Roho” and Seventh Day Adventist religion not permitting fires lit on Saturday and Sunday | (+) Social norm of sharing household responsibilities among women and male and female teenagers  (-) Competing priorities in relation to work and household duties | (-) Some caregivers believed that if specific food were covered, it would go bad, leading to them not covering food |
| **Automatic motivation (wants,**  **needs, automatic responses,**  **and impulses)** | (+) Presence of visible dirt facilitated handwashing for children  (-) Some caregivers did not wash hands in the absence of visible dirt, even though the hands may have been contaminated |  |  | (+) Disgust from flies from latrine landing on unwashed/dirty utensils | (+) Concerns about flies getting into uncovered food |
| **Reflective motivation (self-conscious planning and evaluation, and beliefs about what is good or bad)** | (+) Belief that dirty hands could harbor microbes which can make one sick  (-) Caregivers prioritized water for alternative uses over washing hands | (+) Caregivers prioritized water for washing food | (-) Caregivers prioritized offsite work to coming home to reheating food | (+) Caregivers had a specific routine designated to cleaning utensils  (-) Some caregivers prioritized other work to cleaning utensils | (-) Some caregivers believed that moisture from covering hot food will spoil it |

*Key: (+) – facilitator ( what made one practice behavior) ; (+/-) – facilitator and barrier; (-) – barrier (hindrance to practicing behavior).
